# Durability of ChAdOx1 nCoV-19 vaccination in people living with HIV

**DOI:** 10.1172/jci.insight.157031

**Published:** 2022-04-08

**Authors:** Ane Ogbe, Matthew Pace, Mustapha Bittaye, Timothy Tipoe, Sandra Adele, Jasmini Alagaratnam, Parvinder K. Aley, M. Azim Ansari, Anna Bara, Samantha Broadhead, Anthony Brown, Helen Brown, Federica Cappuccini, Paola Cinardo, Wanwisa Dejnirattisai, Katie J. Ewer, Henry Fok, Pedro M. Folegatti, Jamie Fowler, Leila Godfrey, Anna L. Goodman, Bethany Jackson, Daniel Jenkin, Mathew Jones, Stephanie Longet, Rebecca A. Makinson, Natalie G. Marchevsky, Moncy Mathew, Andrea Mazzella, Yama F. Mujadidi, Lucia Parolini, Claire Petersen, Emma Plested, Katrina M. Pollock, Thurkka Rajeswaran, Maheshi N. Ramasamy, Sarah Rhead, Hannah Robinson, Nicola Robinson, Helen Sanders, Sonia Serrano, Tom Tipton, Anele Waters, Panagiota Zacharopoulou, Eleanor Barnes, Susanna Dunachie, Philip Goulder, Paul Klenerman, Gavin R. Screaton, Alan Winston, Adrian V.S. Hill, Sarah C. Gilbert, Miles Carroll, Andrew J. Pollard, Sarah Fidler, Julie Fox, Teresa Lambe, John Frater

**Affiliations:** 1 Peter Medawar Building for Pathogen Research, Nuffield Dept of Clinical Medicine, and; 2 The Jenner Institute, Nuffield Department of Medicine, University of Oxford, Oxford, United Kingdom.; 3 Department of Infectious Disease, Faculty of Medicine, Imperial College London, London, United Kingdom.; 4 Department of HIV Medicine, St. Mary’s Hospital, Imperial College Healthcare NHS Trust, London, United Kingdom.; 5 Oxford Vaccine Group, Department of Paediatrics, University of Oxford, Oxford, United Kingdom.; 6 NIHR Imperial Clinical Research Facility and NIHR Imperial Biomedical Research Centre, London, United Kingdom.; 7 NIHR Guy’s and St Thomas’ Biomedical Research Centre, London, United Kingdom.; 8 Department of Infection, Harrison Wing and NIHR Clinical Research Facility, Guy’s and St Thomas’ NHS Trust, London, United Kingdom.; 9 Wellcome Centre for Human Genetics, Nuffield Department of Medicine, University of Oxford, Oxford, United Kingdom.; 10 NIHR Oxford Biomedical Research Centre, Oxford, United Kingdom.; 11 Oxford University Hospitals NHS Foundation Trust, Oxford, United Kingdom.; 12 Centre for Tropical Medicine and Global Health, Nuffield Department of Medicine, University of Oxford, Oxford, United Kingdom.; 13 Mahidol-Oxford Tropical Medicine Research Unit, Mahidol University, Bangkok, Thailand.; 14 Department of Paediatrics, University of Oxford, Oxford, United Kingdom.; 15 Public Health England, Porton Down, United Kingdom.

**Keywords:** AIDS/HIV, COVID-19, Adaptive immunity, Cellular immune response, T cells

## Abstract

Duration of protection from SARS-CoV-2 infection in people living with HIV (PWH) following vaccination is unclear. In a substudy of the phase II/III the COV002 trial (NCT04400838), 54 HIV^+^ male participants on antiretroviral therapy (undetectable viral loads, CD4^+^ T cells > 350 cells/μL) received 2 doses of ChAdOx1 nCoV-19 (AZD1222) 4–6 weeks apart and were followed for 6 months. Responses to vaccination were determined by serology (IgG ELISA and Meso Scale Discovery [MSD]), neutralization, ACE-2 inhibition, IFN-γ ELISpot, activation-induced marker (AIM) assay and T cell proliferation. We show that, 6 months after vaccination, the majority of measurable immune responses were greater than prevaccination baseline but with evidence of a decline in both humoral and cell-mediated immunity. There was, however, no significant difference compared with a cohort of HIV-uninfected individuals vaccinated with the same regimen. Responses to the variants of concern were detectable, although they were lower than WT. Preexisting cross-reactive T cell responses to SARS-CoV-2 spike were associated with greater postvaccine immunity and correlated with prior exposure to beta coronaviruses. These data support the ongoing policy to vaccinate PWH against SARS-CoV-2, and they underpin the need for long-term monitoring of responses after vaccination.

## Introduction

The global COVID-19 pandemic has led to over 200 million cases and 4.2 million deaths (1). Vaccines that have been licensed against SARS-CoV-2 include the AstraZeneca ChAdOx1 nCoV-19 (AZD1222) adenoviral vectored vaccine, of which over 1 billion doses have been made available worldwide. People living with HIV (PWH) represent a high-risk group for adverse clinical outcomes from viral infections such as influenza and COVID-19, with some evidence for higher hospitalization and mortality rates (2–6). This can, in part, be attributed to a state of immune cell depletion and chronic immunopathology, including immune activation and exhaustion, which is only partially restored by antiretroviral therapy (ART; refs. 7, 8). Studies on influenza and tetanus toxin vaccination in PWH have shown that antibody levels after vaccination were dependent on CD4 T cell count and activated T follicular helper (Tfh) cell frequencies, which can vary widely in PWH (9, 10), resulting in broader concerns over reduced responses to vaccines (11) and specific vaccination guidelines for PWH (12). Some studies also report that vaccination of PWH may induce immune activation and reactivate the HIV reservoir (13, 14).

ChAdOx1 nCoV-19 containing SARS-CoV-2 full-length spike has been shown to induce potent humoral and cellular immune response in vaccine recipients (15–18). We recently reported the safety and immunogenicity of the ChAdOx1 nCoV-19 vaccine in PWH up to 2 months after initial vaccination (16) and the durability of T and B cell responses following natural infection with SARS-CoV-2 (19). There are, however, few studies evaluating the durability of immunity following vaccination against COVID-19 (20, 21). A recent open label phase I trial showed durable SARS-CoV-2 T and B cell immune response up to 6 months following vaccination in adults without HIV using a low dose of mRNA vaccine mRNA-1273 (21), with similar results in another study using standard mRNA-1273 dosing (22). There have been no studies to date reporting the durability of immune responses in PWH, to our knowledge.

Since the rollout of COVID-19 vaccines, divergent mutations in the viral sequence in the original SARS-CoV-2 strain have given rise to the Alpha (B.1.1.7), Beta (B.1.351), Gamma (P.1), and more recently Delta (B.1.617.2) variants of concern (VOCs). Infections with VOCs have become dominant in several countries (23). Studies of symptomatic disease in fully vaccinated individuals report variable effectiveness (ChAdOx1 nCoV-19, Alpha [74%], Delta [67%]; BNT162b2, Alpha [93.7%], Delta [88%]), with evidence for sustained protection from severe disease (24–26). Nonetheless, breakthrough infections have been recorded, and a significant proportion of the world’s population remains unvaccinated (27). Understanding the ability of immune responses generated in PWH to recognize VOCs is key to informing vaccination strategies, especially in vulnerable populations.

Preexisting cross-reactive T and B cell responses in individuals naive to SARS-CoV-2 infection and vaccination to the circulating common cold coronaviruses (CCC) HKU1, OC43, 299E, and NL63 have been identified (28–34); however, the impact of this cross-reactivity is unclear. While some reports point to a beneficial role in mitigating disease severity and the induction of neutralizing antibodies in both vaccination and natural infection (32, 35, 36), others report no biological function (37, 38) or a potential pathological role (39).

In this open-label, nonrandomized substudy of male participants with HIV on ART (CD4^+^ T cell count > 350 cells/μL) receiving ChAdOx1 nCoV-19, we investigated the immunological landscape 6 months after vaccination. We evaluated the durability of the cellular and humoral immune response to SARS-CoV-2 and VOCs, and we assessed the potential role of cross-reactive CCC immune responses in the modulation of postvaccine responses, presenting evidence for an interaction with the beta coronaviruses, HKU1 and OC43.

## Results

### Participants.

**Figure 1:**
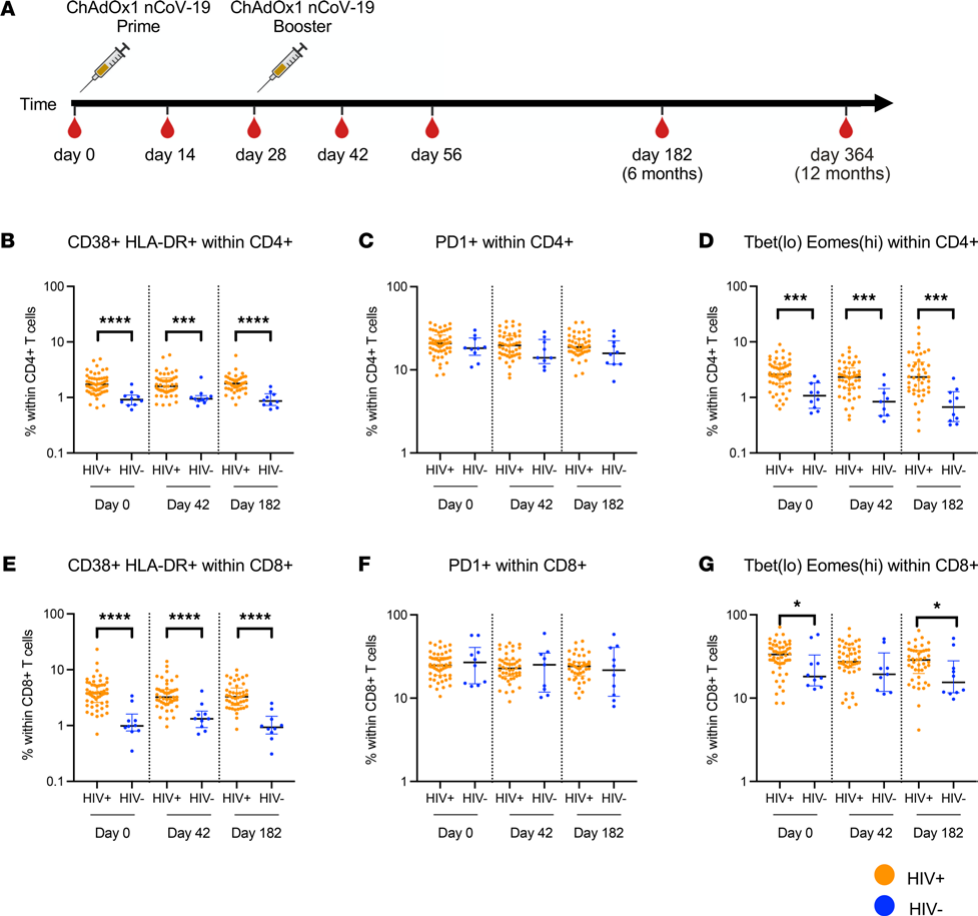
PWH show higher baseline immune activation and exhaustion. (**A**) Schematic showing vaccination schedule for ChAdOx1 nCoV-19 in PWH. (**B**–**G**) Frequency of (**B**) CD38^+^ HLA-DR^+^, (**C**) PD1^+^, and (**D**) Tbet^lo^Eomes^hi^ cells within CD4^+^ and (**E**) CD38^+^ HLA-DR^+^, (**F**) PD1^+^, and (**G**) Tbet^lo^Eomes^hi^ cells within CD8^+^ T cells. Comparison of 2 groups by 2-tailed Mann-Whitney *U* test. *P ≤ 0.05, ***P ≤ 0.001, and ****P ≤ 0.0001. *n =* 48–54 for HIV^+^ volunteers and 10 for HIV^–^ control proliferation assay. Data are shown as median ± IQR.

PWH (*n =* 54; all male) were recruited as part of the ChAdOx1 nCoV-19 COV002 clinical trial (NCT04400838) in November 2020. Participants had undetectable plasma viral load (VL) (<50 HIV RNA copies/mL) and a median CD4 count of 694 cells/μL (IQR, 573.5–859.5) at the time of recruitment. Most participants were White (81.5%). Other reported races were Asian (3.7%), mixed (7.4%), and other (Black, other [missing data], 7.4%). HIV seronegative controls were provided from the ChAdOx1 nCoV-19 COV002 clinical trial. All participants received ChAdOx1 nCoV-19 between 4 and 6 weeks apart and were followed for 6 months (Figure 1A and Table 1).

### Persistent immune activation in PWH before and after vaccination.

T cell immune activation and exhaustion were assayed at day 0 baseline, day 42, and day 182 after first ChAdOx1 nCoV-19 vaccination (Figure 1, B–G). There were significantly higher frequencies of CD38^+^HLA-DR^+^ expressing CD4^+^ and CD8^+^ cells in PWH compared with HIV^–^ controls, consistent across all time points (Figure 1, B and E, and gating strategy in Supplemental Figure 1A; supplemental material available online with this article; https://doi.org/10.1172/jci.insight.157031DS1). There was a transient increase in the frequency of CD38^+^HLA-DR^+^ CD4^+^ and CD8^+^ T cells 14 days after vaccination in PWH, which returned to prevaccination levels by 6 months (Supplemental Figure 1, C and F). Expression of the immune checkpoint inhibitor PD-1 on CD4^+^ and CD8^+^ T cells was not significantly different between PWH and HIV^–^ controls, with no statistically significant changes after vaccination (Figure 1, C and F, and Supplemental Figure 1B). The frequency of CD4^+^ and CD8^+^ PD-1–expressing cells fluctuated early after vaccination in PWH but was restored to prevaccination levels at 6 months (Supplemental Figure 1, D, G, J, and M). The frequency of phenotypically exhausted Tbet^lo^Eomesodermin^hi^ (Tbet^lo^Eomes^hi^) CD4^+^ and CD8^+^ T cells was higher in PWH compared with HIV^–^ individuals both before and after vaccination. (Figure 1, D and G, and Supplemental Figure 1, B, E, H, K, and N).

### Humoral immunity against ChAdOx1 nCoV-19 in PWH persists for 6 months.

**Figure 2:**
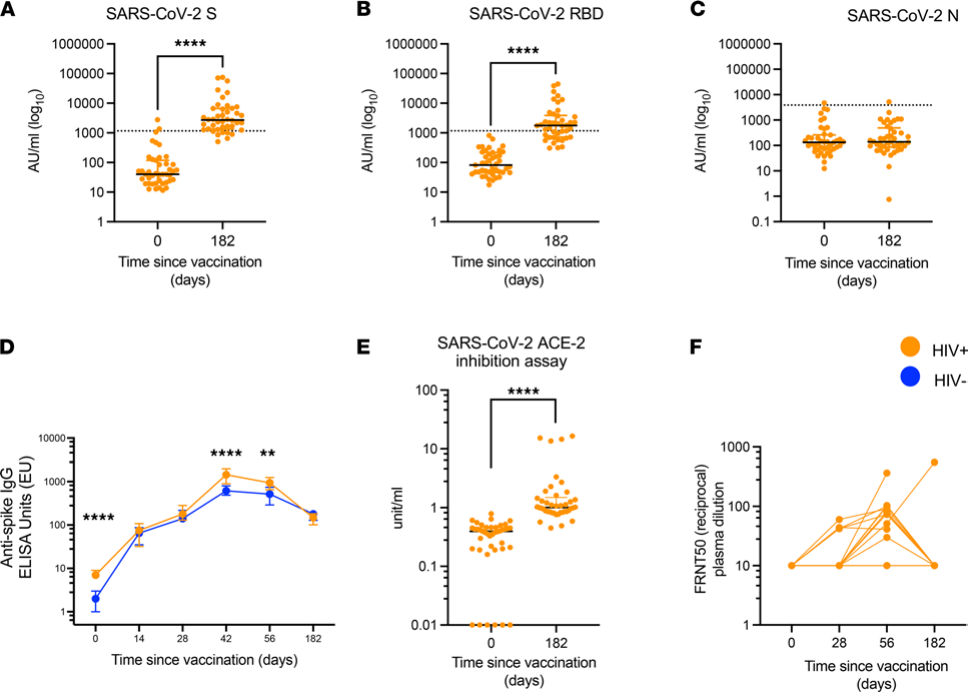
Antibody levels against SARS-CoV-2 six months after ChAdOx1 nCoV-19 vaccination. (**A**–**C**) IgG levels for SARS-CoV-2 (**A**) spike, (**B**) RBD, and (**C**) N protein measured at day 0 (baseline) and day 182 (6 months after vaccination) using the MSD ELISA assays. (**D**) Comparison between antibody kinetics in HIV^+^ and HIV^–^ across all available time points. (**E** and **F**) ACE-2 inhibition assay at baseline and 6 months after vaccination and live-virus focus reduction neutralization assay (FRNT) on *n* = 15 HIV^+^ donors on days 0, 28, 56 and 182. Comparison of 2 time points within the same group was done by Wilcoxon matched-pairs signed-rank test. Comparison of 2 groups was done by Mann-Whitney *U* test with Bonferroni-Dunn’s multiple-comparison test (Prism v9. **B** shows adjusted significant levels. **P ≤ 0.01 and ****P ≤ 0.000. Dotted lines in **A**–**C** indicate cut-off points determined for each SARS-CoV-2 antigen (S, RBD, and N) based on prepandemic sera + 3 SD. *n =* 42–54 for HIV^+^ volunteers in MSD assay, in-house ELISA, and ACE-2 inhibition assay; 14–15 in FRNT assays; and 54 for HIV^–^ controls. Data are shown as median ± IQR.

We previously reported detectable antibody levels up to 56 days following ChAdOx1 nCoV-19 vaccination in PWH (16). To determine the further persistence of antibody responses, total IgG for spike (S), receptor binding domain (RBD) and nucleocapsid (N), as well as neutralizing antibody levels, were measured at days 0 and 182. Two independent ELISA technologies were used for binding IgG assays: a standardized in-house total IgG against spike and Meso Scale Discovery (MSD) binding assays measuring S, RBD, and N antibody levels. Levels of anti–spike IgG measured using the 2 assays were positively correlated (Supplemental Figure 2, A and B; *r* = 0.7, *P <* 0.0001, and *r* = 0.9, *P <* 0.0001, at days 0 and 182, respectively; Spearman’s rank). At day 182 after vaccination, antibodies to S and RBD but not N were significantly higher than at baseline (S, day 0 = 3/43 participants [6.9%], day 182 = 35/42 participants [83.3%]; RBD, day 0 = 0/43 participants [0%], day 182 = 27/42 participants [64.2%]) (Figure 2, A–C), consistent with observed responses being driven by vaccination rather than infection.

Importantly, there was no difference in total anti-spike antibody titres in HIV^+^ and HIV^–^ matched participants measured at 182 days after first vaccination, although with some waning of responses in both groups after day 56 (Figure 2D and Supplemental Figure 2, D and E). Prevaccine baseline antibody titres correlated positively with early postvaccination time points at days 14 and 28 but not days 42, 56, or 182 (Supplemental Figure 2F and Supplemental Table 1).

We next assessed the ability of antibodies from plasma collected 6 months after vaccination to compete with SARS-CoV-2 for binding to ACE-2 using an ACE-2 inhibition assay and to neutralize SARS-CoV-2 using a live virus focus reduction neutralization assay (FRNT). FRNT was performed in a randomly selected subset of the cohort for whom we have previously reported neutralization antibody levels up to day 56 (16). At day 182 after ChAdOx1 nCoV-19 prime, antibodies capable of blocking the SARS-CoV-2 ACE-2 interaction were present at significantly higher levels than at prevaccination baseline (Figure 2E) and correlated strongly with anti-RBD antibodies (Supplemental Figure 2C). However, at the same time point, antibody neutralization measured by FRNT live virus assay revealed titres below the assay detection limit in nearly all participants (13 of 14; 92%; Figure 2F).

### Durable SARS-CoV-2–specific T cell responses are induced following ChAdOx1 nCoV-19 vaccination.

**Figure 3:**
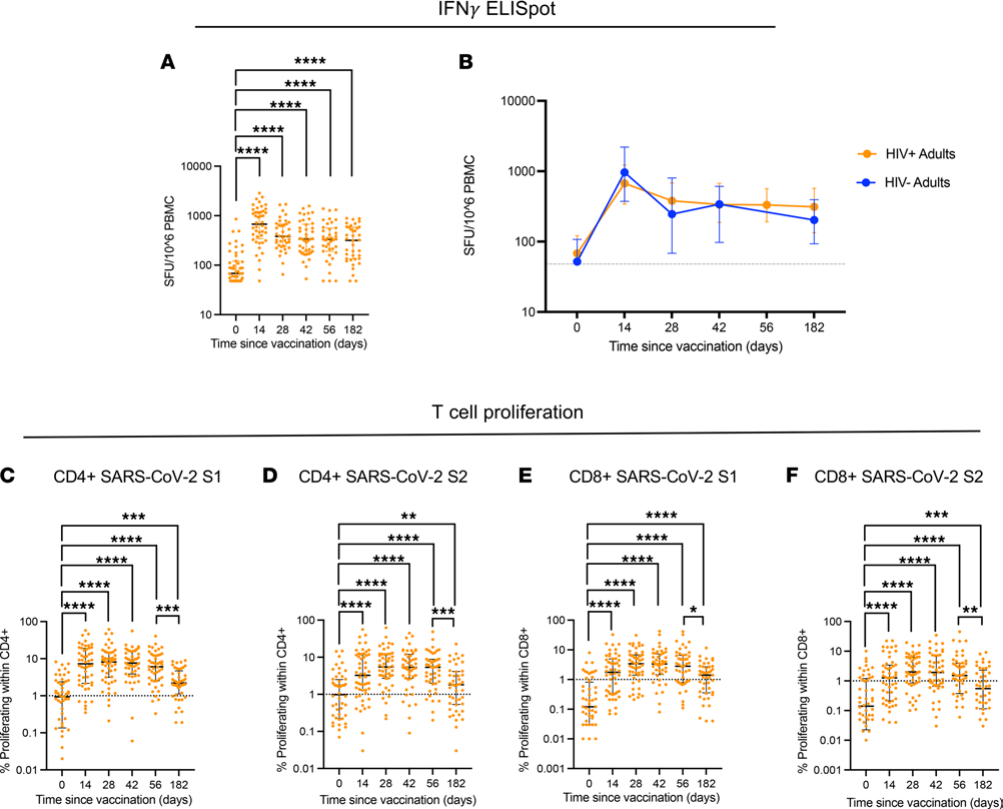
T cell responses following ChAdOx1 nCoV-19 vaccination are durable in PWH. (**A**) T cell response measured using peptides pools against SARS-CoV-2 S1 and S2 antigens by IFN-γ ELISpot across all time points. (**B**) Comparative analysis of IFN-γ T cell responses in HIV^+^ and HIV^–^ volunteers. (**C** and **D**) Proliferative T cell responses to (**C**) SARS-CoV-2 S1 and (**D**) SARS-CoV-2 S2 in CD4^+^ T cells across all available time points. (**E** and **F**) Proliferative T cell responses to (**E**) SARS-CoV-2 S1 and (**F**) SARS-CoV-2 S2 in CD8^+^ T cells across all available time points. Comparison of 2 time points within the same group was done by Wilcoxon matched-pairs signed-rank test. Comparison of 2 groups was done by 2-tailed Mann-Whitney *U* test or multiple Mann-Whitney *U* test (**B**) with Bonferroni-Dunn’s multiple-comparison test (Prism v9). *P ≤ 0.05, **P ≤ 0.01, ***P ≤ 0.001, and ****P ≤ 0.000. Dotted lines in **C**–**F** indicate threshold for true positive based mean of DMSO controls + 3 SD. *n =* 48–54 for HIV^+^ volunteers and 54 for HIV^–^ controls. Data are shown as median ± IQR.

Durability of vaccine-induced SARS-CoV-2–specific T cell immunity at 6 months was assessed by IFN-γ ELISpot and T cell proliferation assays. SARS-CoV-2 spike-specific ELISpot responses were maintained for 6 months in PWH following vaccination and were equivalent to the HIV^–^ control group (Figure 3, A and B, and Supplemental Figure 4A).

For further resolution of the durability of T cell immunity, we used a T cell proliferation assay, which also allows distinction of different CD4^+^ and CD8^+^ T cell lineage responses. The spike peptide pool was separated into S1 and S2. Gating strategy is shown in Supplemental Figure 3A. The frequency of SARS-CoV-2 spike-specific proliferative CD4^+^ and CD8^+^ T cell responses in PWH following vaccination were maintained at levels significantly higher than at baseline for 6 months (Figure 3, C–F). Longitudinal responses to FECT controls remained unchanged, while PHA responses were back to baseline by day 182 (Supplemental Figure 3, B and C). There was no difference in the magnitude of the vaccine-specific T cell proliferative responses between the HIV^+^ and the HIV^–^ cohorts (Supplemental Figure 4, F–L). Although T cell responses in PWH measured by IFN-γ ELISpot peaked at day 14 and were then maintained to day 182, proliferative responses peaked later at day 42 and then contracted, such that day 182 responses were significantly lower than those measured at day 56 (Figure 3, C–F). These kinetics are similar to those observed with the anti-S antibody response (Supplemental Figure 2, D and E).

### Vaccine-reactive T cells are not differentially biased to a specific CD4^+^ subset.

**Figure 4:**
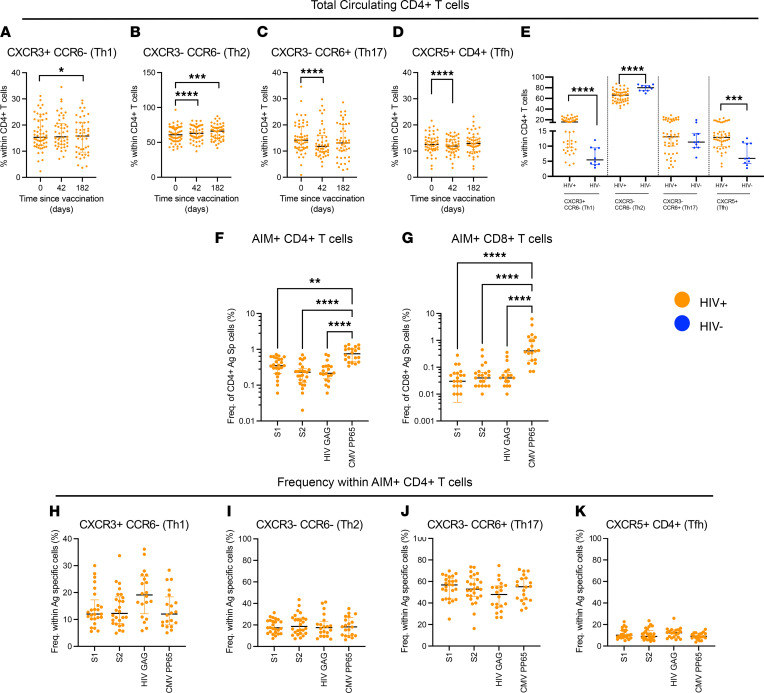
SARS-CoV-2–specific T cells are not preferentially biased for any CD4^+^ T cell subsets. (**A**–**D**) Ex vivo frequencies of (**A**) CXCR3^+^CCR6^–^ (Th1), (**B**) CXCR3^–^CCR6^–^ (Th2), (**C**) CXCR3^–^CCR6^+^ (Th17), and (**D**) CXCR5^+^ within CD4^+^ T cells in HIV^+^ volunteers measured at days 0, 42, and 182 using ex vivo T cell phenotyping. (**E**) Comparative analysis of frequencies of ex vivo CD4^+^ T cell frequencies in HIV^+^ and HIV^–^ volunteers at day 182 (6 months after vaccination). (**F** and **G**) Measurement of frequencies of antigen-specific T cells including SARS-CoV-2 S1 and S2, HIV gag, and CMVpp65 using activation-induced marker (AIM) assay in (**F**) CD4^+^ and (**G**) CD8^+^ T cells. Using ‘or’ Boolean gating on FlowJo, antigen specific CD4^+^ T cells were: CD25^+^CD134(OX40)^+^, CD25^+^CD137^+^, or CD25^+^CD69^+^; for CD8^+^ T cells, antigen specific cells were: CD25^+^CD137^+^ or CD25^+^CD69^+^. (**H**–**K**) Frequencies of (**H**) CXCR3^+^ CCR6^–^ (Th1), (**I**) CXCR3^–^CCR6^–^ (Th2), (**J**) CXCR3^–^CCR6^+^ (Th17), and (**K**) CXCR5^+^ CD4^+^ T cells within antigen-specific (AIM^+^) T cells in HIV^+^ volunteers. Comparison of 2 time points within the same group was done by Wilcoxon matched-pairs signed-rank test. Comparison of 2 groups was done by 2-tailed Mann-Whitney *U* test. *P ≤ 0.05, **P ≤ 0.01, ***P ≤ 0.001, and ****P ≤ 0.000. *n =* 48 – 54 for HIV^+^ volunteers in ex vivo phenotyping assay, 20 for HIV^+^ volunteers in AIM assay, and 10 for HIV^–^ control in ex vivo phenotyping assay. Data are shown as median ± IQR.

Using CCR6, CXCR3, and CXCR5 expression to quantify Th1, Th2, Th17, and Tfh cells, we interrogated the phenotype of circulating T cells following vaccination (gating strategy in Supplemental Figure 1A). At 6 months after ChAdOx1 nCoV-19 vaccination, we found redistributions in the phenotype of the CD4^+^ T cells in HIV^+^ volunteers with increases in Th1 (CXCR3^+^CCR6^–^) and Th2 (CXCR3^–^CCR6^–^; Figure 4, A and B) but not Th17 (CXCR3^–^CCR6^+^) or Tfh (CXCR5^+^CD4^+^; Figure 4, C and D). None of these populations correlated with anti-spike antibody levels 6 months after infection. Although the hierarchy in cellular composition of the CD4^+^ T cell subsets was similar in the HIV^+^ and HIV^–^ cohorts, we found circulating frequencies of Th2 subsets to be reduced while Th1 and Tfh subsets were significantly increased 6 months after vaccination in PWH (Figure 4E and Supplemental Figure 5A, for HIV^–^ control data).

The activation induced marker (AIM) assay was used to determine the phenotype of vaccine-specific CD4^+^ T cells 6 months after ChAdOx1 nCoV-19 vaccination (gating strategy in Supplemental Figure 5B). Vaccine responses were compared with concurrent HIV Gag and CMV responses (Figure 4, F and G). Although AIM^+^ cells for all antigens tested showed a Th17 bias (Supplemental Figure 5C), similar to HIV-Gag or CMVpp65-specific T cells, there was no preferential skewing of the SARS-CoV-2–specific T responses to any CD4 Th subset 6 months after vaccination (Figure 4, H–K).

### Responses to VOCs are preserved 6 months after vaccination.

**Figure 5:**
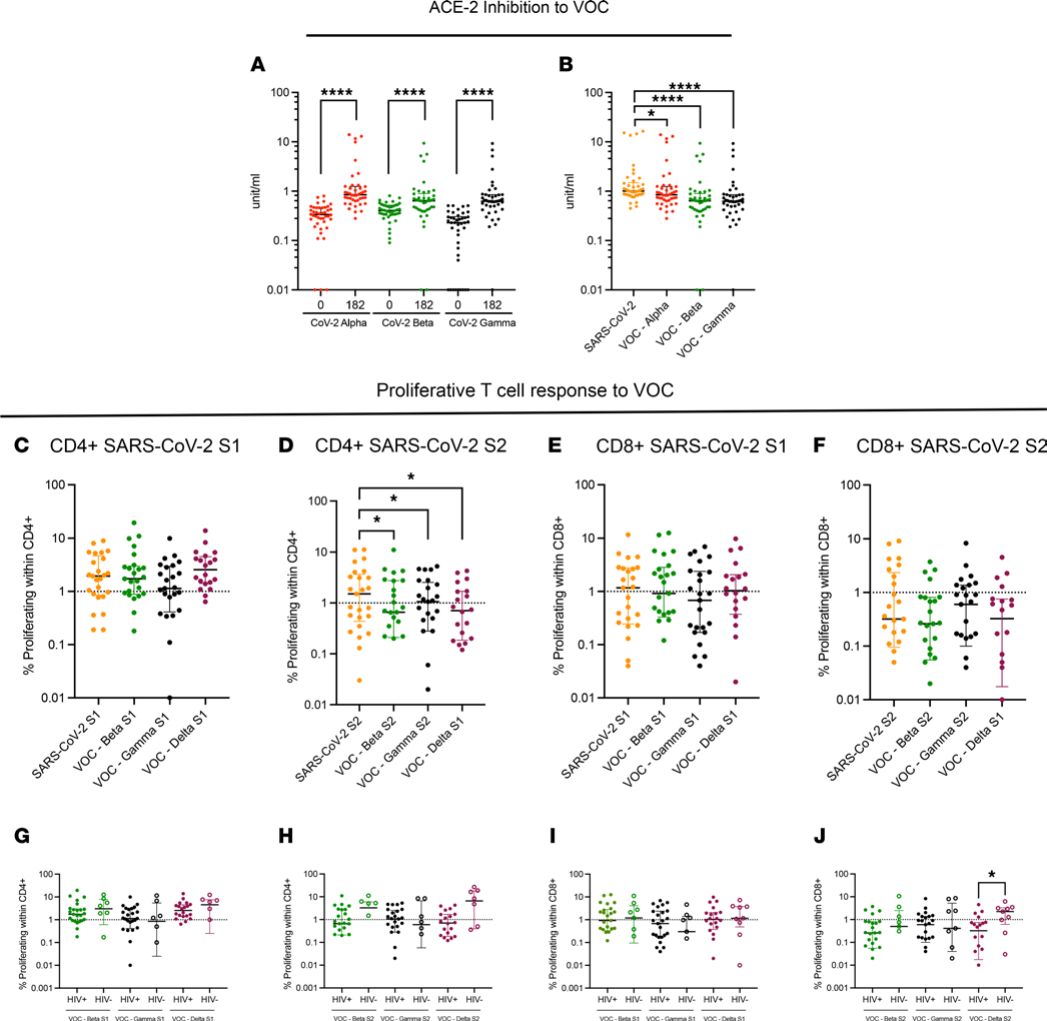
Responses to VOCs are preserved at 6 months after ChAdOx1 nCoV-19 vaccination in PWH. (**A**) ACE-2 binding inhibition assay for Alpha, Beta, and Gamma VOCs measured at day 0 (baseline) and at day 182 (6 months after vaccination) in HIV^+^ volunteers. (**B**) Comparison between ACE-2 binding inhibition of SARS-CoV-2 WT strain and Alpha, Beta, and Gamma VOCs in HIV^+^ volunteers. (**C**–**F**) Comparison between proliferative T cell responses to SARS-CoV-2 WT strain and Beta, Gamma, and Delta VOCs in (**C**) CD4^+^ S1, (**D**) CD4^+^ S2, (**E**) CD8^+^ S1, and (**F**) CD8^+^ S2 in HIV^+^ volunteers. (**G**–**J**) Comparative analysis of (**G**) CD4^+^ S1, (**H**) CD4^+^ S2, (**I**) CD8^+^ S1, and (**J**) CD8^+^ S2 T cells responses to VOCs in HIV^+^ (solid circles) and HIV^–^ (open circles). Comparison of 2 time points within the same group was done by Wilcoxon matched-pairs signed-rank test. Comparison of 2 groups was done by 2-tailed Mann-Whitney *U* test. Where indicated *P ≤ 0.05 and ****P ≤ 0.000. Dotted lines in **C**–**J** indicate threshold for true positive based mean of DMSO controls + 3 SD. *n =* 48–54 for ACE-2 inhibition assay in HIV^+^ volunteers, 20 for HIV^+^ VOC proliferative responses, and 10 for HIV^–^ control VOC responses in proliferation assay. Data are shown as median ± IQR.

Humoral and cellular immune responses to the major VOCs were measured 6 months after vaccination. Inhibition of ACE-2 binding for Alpha, Beta, and Gamma variants was increased compared with prevaccination baseline (Figure 5A); however, there was statistically significant reduction in ACE-2 inhibition for all 3 VOCs compared with the original SARS-CoV-2 strain, which was more apparent in the Beta and Gamma variants (Figure 5B). T cell proliferative responses to VOCs were comparable with the SARS-CoV-2 original strain, except for SARS-CoV-2 CD4 responses to S2, which were moderately reduced across all VOCs tested (Figure 5, C–F). HIV^+^ and HIV^–^ participants had similar magnitudes of T cell responses to S1 and S2 spike proteins of all VOCs tested, with the exception of the CD8^+^ SARS-CoV-2 T cell proliferative response targeting the S2 protein of the Delta variant, which showed a modest reduction in HIV^+^ participants compared with HIV^–^ controls (Figure 5, G–J).

### Modulation of ChAdOx1 nCoV-19 postvaccination responses by preexisting cross-reactive immunity.

SARS-CoV-2 reactive T and B cells exist in unvaccinated COVID-19 naive individuals (Figure 3, A–E, and Supplemental Figure 2, D and E). To determine whether these prevaccine responses might reflect cross-reactivity to endemic circulating coronaviruses of the Alpha (NL63 and 299E) or Beta (HKU1, OC43) genera, we also measured responses to these viruses at baseline.

**Figure 6:**
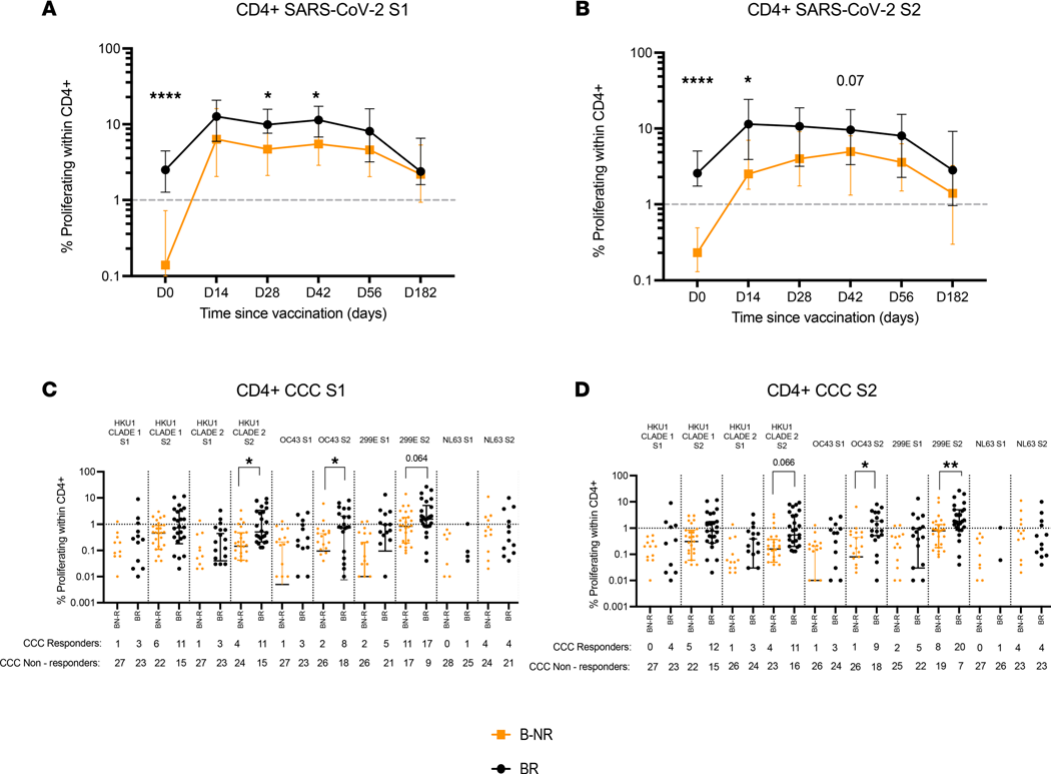
Preexisting cross-reactive CD4^+^ T cell responses in PWH measured at baseline are associated with high-magnitude T cell responses after ChAdOx1 nCoV-19 vaccination. (**A** and **B**) Baseline CD4^+^ SARS-CoV-2 responses were split into baseline responders (BR, proliferation > 1%, black circles and black lines) and baseline nonresponders (B-NR, proliferation < 1%, yellow circles and yellow lines), and CD4^+^ T cell responses after vaccination were analyzed at all available time points for (**A**) SARS-CoV-2 S1 and (**B**) SARS-CoV-2 S2. (**C** and **D**) T cells responses targeting (**C**) S1 and (**D**) S2 proteins in endemic CCCs are measured at baseline in BR and B-NR. Comparison of 2 time points within the same group was done by Wilcoxon matched-pairs signed-rank test. Comparison of 2 groups was done by 2-tailed Mann-Whitney *U* test with Bonferroni-Dunn’s multiple-comparison test (Prism v9). **A** and **B** show adjusted significant levels. CCC responses among participants were compared using Fisher’s exact test and listed in Supplemental Table 3. *P* values as indicated or *P ≤ 0.05, **P ≤ 0.01, and ****P ≤ 0.000. Dotted lines indicate threshold for true positive based mean of DMSO controls + 3 SD. *n =* 48–54 for HIV^+^ volunteers. Data are shown as median ± IQR.

Based on the T cell proliferation assay, participants were divided according to those with prevaccine baseline SARS-CoV-2 immune responses (baseline responders [BR]) and those without preexisting immunity (baseline nonresponders [B-NR]). Regardless of any preexisting immunity, all donors mounted an immune response following vaccination; however, BR consistently showed higher magnitude CD4^+^ (Figure 6, A and B) and CD8^+^ T cell (Supplemental Figure 6, A and B) responses to SARS-CoV-2 S1 and S2 at most postvaccination time points. Baseline SARS-CoV-2 CD4^+^ S2 (and, to a lesser extent, S1) T cell proliferation was positively correlated with subsequent postvaccine proliferative responses targeting the same regions (Supplemental Figure 7, B and C, and Supplemental Table 2, A–D), which is of potential interest, as S2 is associated with regions of homology to other coronaviruses.

**Figure 7:**
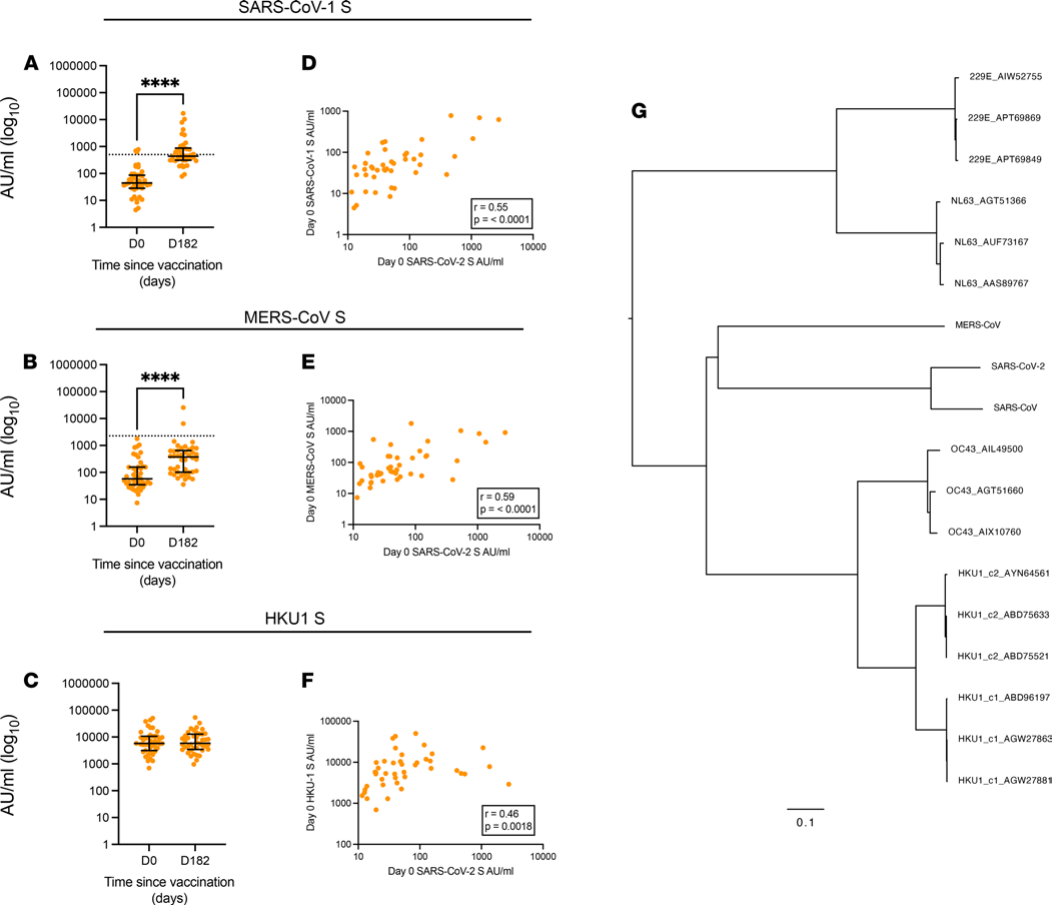
Cross-reactive humoral immune responses among Beta CoVs. (**A**–**C**) Antibody titres against (**A**) SARS-CoV, (**B**) MERS-CoV, and (**C**) HKU1 spike proteins measured at day 0 (baseline) and day 182 (6 months after vaccination) in HIV^+^ participants. (**D**–**F**) Correlation between baseline antibody titres for SARS-CoV-2 and (**D**) SARS-CoV-1, (**E**) MERS-CoV, and (**F**) HKU1 spike protein at baseline. (**G**) Phylogenetic tree showing relationship between coronaviruses. Correlation was performed via Spearman’s rank correlation coefficient, and comparison of 2 time points within the same group was done by Wilcoxon matched-pairs signed-rank test. ****P ≤ 0.0001. Dotted lines in **A** and **B** indicate cut-off points determined for each SARS-CoV-2 antigen based on prepandemic sera + 3 SD. *n =* 48–54 for HIV^+^ volunteers. Data are shown as median ± IQR.

T cell and IgG responses to the endemic CCCs (HKU1 [clade 1 and 2], OC43, 299E, and NL63) in HIV-infected participants remained mostly unchanged by vaccination with ChAdOx1 nCoV-19, indicating that vaccination did not boost these responses (Figure 7C and Supplemental Figures 8 and 9); however, IgG responses to SARS-CoV-1 and MERS-CoV in PWH were higher at 6 months (Figure 7, A and B).

Focusing on baseline preexisting responses — and dividing the cohort of PWH into the SARS-CoV-2 BR and B-NR groups as before — participants with baseline proliferative T cell responses to SARS-CoV-2 spike also had T cell responses targeting the S2 spike regions of CCCs, especially for the Beta coronaviruses HKU1 and OC43 and Alpha coronavirus 299E (Figure 6, C and D; Supplemental Figure 6, C and D; and Supplemental Table 3). This was supported by humoral responses taken at the same prevaccination time point, which showed strong correlations between SARS-CoV-2 spike IgG levels and those of SARS-CoV-1, MERS-CoV-1, and HKU1 (Figure 7, D–F; Supplemental Figure 9; and Supplemental Table 4). Phylogenetic analysis of spike sequences showed that OC43 and HKU1 were the mostly closely related CCCs to SARS-CoV-2 (Figure 7G). These data suggest that prior exposure to Beta coronaviruses and responses to the S2 homologous region may potentially be associated with larger and more persistent T cell responses following SARS-CoV-2 vaccination.

## Discussion

Long-lasting immune responses against SARS-CoV-2 will be necessary to confer protection from severe COVID-19. Although clinical management and effective ART have improved long-term outcomes for PWH — especially in resource-rich countries — immunopathology in HIV-infected subjects, evidenced by increased immune activation and exhaustion, remains significantly high compared with the levels found in HIV-uninfected counterparts (7, 40–42), raising concerns whether effective immune responses will persist after vaccination. We show here for the first time to our knowledge in PWH that vaccine-induced immunity to SARS-CoV-2 persists for at least 6 months by most assays, but with evidence that responses are starting to wane. There were no significant differences in responses by PWH and HIV^–^ controls, extending the data from short-term responses reported previously (16, 43). Study participants were predominantly White males with well-controlled HIV on ART; therefore one should be cautious extrapolating these data to other populations. However, recent data from the USA, Chile, and Peru show no significant impact of sex or race/ethnicity on efficacy of the AZD1222 vaccine (44).We confirm the persistent immune activation — and, to a lesser degree, phenotypic exhaustion — in T cells in PWH on ART, but we show that this does not impact the robust humoral and cellular immune responses to ChAdOx nCoV-19 that persist for 6 months. Reports on reactivation of the HIV reservoir and increased immune activation after vaccination in PWH are conflicting (13, 14, 45), and although we found a transient increase in the frequencies of T cells coexpressing CD38 and HLA-DR, this was restored to baseline by 6 months. Further studies will be needed to determine any impact on the HIV reservoir.

Vaccine design and regimen can skew the quality of the T cell response by the preferential induction of one CD4^+^ Th subset over another (46–51). ChAdOx-1 nCoV-19 responses show a qualitative skew toward the Th1 phenotype, with increased IFN-γ–, IL-2–, and TNF-producing T cells shortly after vaccination (18). Other studies in convalescent cohorts have linked a CCR6^+^ Th17 Tfh cell phenotype with reduced disease severity (52). Similar to others (47, 53), we found antigen-specific CD4^+^T cells following vaccination were mostly a CCR6^+^CXCR3^–^ Th17 phenotype. We did not find SARS-CoV-2 spike-specific CD4^+^ T cells biased toward any chemokine-expressing subpopulation 6 months after vaccination, possibly reflecting the longer duration between vaccination and analysis than in other studies. Similar to other studies (47, 53), we defined these circulating Th subsets using CXCR3 and CCR6 chemokine receptors. The resulting population based on these 2 markers alone may not be homogenous for 1 Th subset. Inclusion of other chemokine receptors such as CCR3, CCR4, and CCR8 (54–57) to further delineate Th subsets can provide a deeper resolution of the Th bias in total and antigen-specific Th subsets following ChAdOx nCoV-19 vaccination.

Understanding durability of both humoral and cellular immunity to SARS-CoV-2 — both likely key components of an effective response (52, 58, 59) — is key to understanding long-term protection. When we assessed the longevity of the humoral and cellular immunity in PWH 6 months after ChAdOx1 nCoV-19 vaccination, we found that vaccine-mediated antibodies to spike or RBD remained elevated above baseline and were no different from HIV^–^ controls. Similarly, T cell responses to spike were maintained at magnitudes above baseline and demonstrated similar kinetics to HIV^–^ participants. Antibody function measured by ACE-2 binding inhibition was sustained at levels above prevaccination; however, live neutralization assays did not detect antibodies in the majority of the participants assayed at 6 months. Both assays identified the same participants as low (*n =* 13) and high (*n =* 1) responders, and the ACE-2 binding inhibition and SARS-CoV-2 RBD titres showed a strong positive correlation. We speculate that, although differences in positive responses between the 2 functional assays could be as a result of function (neutralization) versus antigenicity (ACE-2 binding inhibition), it could also, in part, be due to assay sensitivity and differing dynamic ranges between assays.

SARS-CoV-2 convalescent plasma has been shown to have effective FC-mediated antibody functions such as antibody-dependent cellular phagocytosis (ADCP), antibody-dependent cellular cytotoxicity (ADCC), and complement-dependent cytotoxicity (CDC; refs. 60–62), which are more durable than neutralization (61). Nonneutralizing functions were not evaluated in this study; therefore, we cannot exclude that these are preserved in this cohort of PWH. Total spike IgG antibody and T cell proliferative responses in PWH were significantly lower at 6 months after vaccination compared with day 56. These results suggest detectable but waning T and B cell responses at 6 months. Similar findings were reported for the mRNA-1273 COVID-19 vaccine and were found to be age-dependent, pointing to immune aging as a contributing factor (20, 21). This comprehensive analysis of humoral and cellular immunity is consistent with studies of COVID-19 in healthy adults and PWH showing durable immune responses up to 7 months after infection (19, 59, 63, 64). Further followup at 12 months and beyond will be important to determine the longer-term persistence of responses, especially when considering the value of booster doses. Furthermore, our study used a short interval between initial and booster dose of 4–6 weeks. Current government policy for the United Kingdom and many other countries advise approximately 8–12 weeks between doses (65); future studies addressing the impact of longer dosing interval on durability of the immune response would be important.

The emergence of VOCs poses a potential roadblock to ending the pandemic. We found humoral immunity to VOCs at 6 months to be at titres lower than those targeting the original WT SARS-CoV-2 strain, albeit still significantly higher than prevaccination levels. The magnitude of the T cell responses to VOCs was similar to those targeting the WT SARS-CoV-2 strain for most VOCs tested, apart from the CD4^+^ S2 responses. For most of the VOCs, T cell responses in PWH did not differ from HIV^–^ controls. Similar observations regarding humoral immunity have been made with the mRNA vaccine BNT162b2, although as most of these studies were done within 2 months of vaccination, information on durability of the response is lacking (66–68). One study assessing T cell responses between 21 and 28 days after full BNT162b2 vaccination found no differences between WT and VOC CD4 responses (66). This study utilized a pool of spike peptide pools not parsed into its S1 and S2 regions, and only a limited panel of VOCs were analyzed. Importantly, emerging data from real-world effectiveness studies suggest that vaccination protects against death and severe disease, even following infection with VOCs (24, 26)

Cross-reactivity from previous CCC infection may impact the measured SARS-CoV-2 immune response after vaccination and natural infection (31, 32, 35). We identified measurable prevaccine antibody titres for SARS-CoV-2 S, RBD, and N proteins in PWH. Prevaccination SARS-CoV-2 S antibody levels strongly correlated with those of contemporaneous Beta coronaviruses SARS-CoV-1, MERS-CoV, and HKU1 (of which only the latter is likely to have been experienced by these United Kingdom study participants), supporting the hypothesis that these titres result from previous infection with a similar coronavirus and some cross-reactivity across coronaviruses. Supporting the antibody data, the presence of cross-reactive T cells before vaccination (based on proliferative potential following antigen challenge) was associated with higher-magnitude postvaccination T cell responses.

There is much debate over the significance of cross-reactive responses. Studies have reported reduced disease severity in patients with CCC humoral responses and regions of high homology to CCC capable of transpriming SARS-CoV-2 T and B cell responses (31, 32, 35). Preexisting immunity was also shown to boost postvaccine responses in low dose mRNA-1273 vaccine (21), although an explanatory mechanism was not reported. Further investigations in large studies would be needed to fully elucidate the impact of baseline preexisting immunity in postvaccination response, but we have found clear evidence of higher-magnitude immune responses in those with cross-reactivity. Although our data suggest that responses to CCC may help augment subsequent vaccine responses against SARS-CoV-2, we have no evidence that, on their own, they are potent enough to impact susceptibility to COVID-19.

In summary, we present a comprehensive immunological assessment of ChAdOx1 nCoV-19 in PWH 6 months after vaccination. We show that, despite persistent immune activation in PWH, PWH on ART and HIV-uninfected participants make equivalent T and B cell responses following vaccination. However, both responses showed signs of decline after 6 months. It is unknown what level of immunity is required to prevent hospitalization and mortality, but real-world data suggest vaccination is successful in preventing severe disease and death even in the presence of transmissible and virulent VOC (24, 26). A booster dose may become necessary in the future to maintain long-term immunological memory to SARS-CoV-2 and the VOCs, especially for susceptible cohorts, and we must continue to carefully monitor this going forward. Finally, we demonstrate that preexisting SARS-CoV-2 cross-reactive immune responses to the Beta coronaviruses HKU1 and, to a lesser extent, OC43 are associated with higher-magnitude T cell responses following vaccination in PWH. Together, these data continue to reinforce the policy of ensuring all PWH are offered vaccination against SARS-CoV-2.

## Methods

### Study design and cohort.

The cohort studied in this analysis has been described previously (16). Briefly, the study comprised PWH in an open-label nonrandomized group within the larger multicentre phase II/III COV002 trial. The participants in this single-arm group comprised individuals with HIV who were stable on ART under routine follow-up at 2 London UK National Health Service (NHS) clinics and received ChAdOx1 nCoV-19 vaccination according to the schedule of attendance. Recruitment was done in HIV clinics at 2 centers in the United Kingdom (Imperial College NHS Trust and Guy’s and St Thomas’ NHS Foundation Trust). Inclusion criteria included: age 18–55 years, a diagnosis of HIV infection, virological suppression on ART at enrolment (plasma HIV viral load < 50 copies/mL), and a CD4 count of more than 350 cells/μL. The inclusion criteria for the COV002 trial have been published in full elsewhere (15).

The ChAdOx1 nCoV-19 vaccine was produced as previously described (17). Participants received 2 standard intramuscular doses 4–6 weeks apart. For some assays and where sample availability allowed, comparison was made with age- and sex-matched participants who were HIV^–^, aged 18–55 years, enrolled into the main COV002 phase II/III randomized clinical trial, and randomly assigned (5:1) to receive either ChAdOx1 nCoV-19 or MenACWY by i.m. vaccination. The dose of vaccine administered was the same across both groups. Only participants receiving the ChAdOx1 nCoV-19 vaccine were used for comparison. Full details of the COV002 HIV^–^ cohort have been published previously (15).

A screening visit where a full medical history, examination of all participants, and blood tests to exclude biochemical or hematological abnormalities (full blood count; kidney and liver function tests) was done prior to enrolment. Participants with a history of laboratory-confirmed SARS-CoV-2 infection by anti–N protein IgG immunoassay (Abbott Architect) at screening were excluded. For this study, visits on days 0 (vaccine prime) and 182 were the main study time points used for immunological analysis; however, for some assays, other study visits — days 14, 28 (vaccine boost), 42, and 56 — are presented where available. As some participants did not attend for their day 182 visit (*n* = 6), there is a maximum of *n* = 48 at this time point.

### MSD binding assays.

IgG responses to SARS-CoV-2, SARS-CoV-1, MERS-CoV and seasonal coronaviruses were measured using a multiplexed MSD immunoassay. The V-PLEX COVID-19 Coronavirus Panel 3 (IgG) Kit (catalog K15399U) from Meso Scale Diagnostics. A MULTI-SPOT96-well, 10-spot plate was coated with 3 SARS CoV-2 antigens (S, RBD, N), SARS-CoV-1 and MERS-CoV spike trimers, and spike proteins from seasonal human coronaviruses — HCoV-OC43, HCoV-HKU1, HCoV-229E, and HCoV-NL63 — and bovine serum albumin. Antigens were spotted at 200–400μg/mL (MSD Coronavirus Plate 3). Multiplex MSD assays were performed as per the instructions of the manufacturer. To measure IgG antibodies, 96-well plates were blocked with MSD Blocker A for 30 minutes. Following washing with washing buffer, samples diluted 1:1,000 to 10,000 in diluent buffer, or MSD standard or undiluted internal MSD controls, were added to the wells. After a 2-hour incubation and a washing step, detection antibody (MSD SULFO-TAG anti-human IgG antibody, 1/200) was added. Following washing, MSD GOLD Read Buffer B was added, and plates were read using a MESO SECTOR S 600 Reader. The standard curve was established by fitting the signals from the standard using a 4-parameter logistic model. Concentrations of samples were determined from the electrochemiluminescence signals by back-fitting to the standard curve and multiplied by the dilution factor. Concentrations are expressed in arbitrary units/mL (AU/mL). Cut-offs were determined for each SARS-CoV-2 antigen (S, RBD, and N) based on the concentrations measured in 103 prepandemic sera + 3 SD. Cut-off for S was 1160 AU/mL, cut-off for RBD was 1169 AU/mL, and cut-off for N was 3874 AU/mL.

### SARS CoV-2 spike IgG ELISA.

Humoral responses at baseline and following vaccination were assessed using a standardized total IgG ELISA against trimeric SARS CoV-2 spike protein as described previously (17). In brief, ELISA plates were coated with 2 μg/mL of full-length trimerized SARS-CoV-2 spike glycoprotein and stored at 4°C overnight for at least 16 hours. After coating, plates were washed 6 times with PBS/0.05% Tween and blocked with casein for 1 hour at room temperature (RT). Thawed samples were treated with 10% Triton X-100 for 1 hour at RT and subsequently diluted in casein and plated in triplicate for incubation for 2 hours at RT alongside 2 internal positive controls (controls 1 and 2) to measure plate to plate variation. Control 1 was a dilution of convalescent plasma sample, and control 2 was a research reagent for anti-SARS-CoV-2 Ab (code 20/130 supplied by National Institute for Biological Standards and Control [NIBSC]). The standard pool was used in a 2-fold serial dilution to produce 10 standard points that were assigned arbitrary ELISA units (EUs). Goat anti–human IgG (γ-chain specific) conjugated to alkaline phosphatase was used as secondary antibody, and plates were developed by adding 4-nitrophenyl phosphate in diethanolamine substrate buffer (A3187-1ML, MilliporeSigma). An ELx808 microplate reader (BioTek Instruments) was used to provide optical density measurement of the plates at 405 mm. Standardized EUs were determined from a single dilution of each sample against the standard curve, which was plotted using the 4-parameter logistic model (Gen5 v3.09, BioTek). Each assay plate consisted of samples and controls plated in triplicate, with 10 standard points in duplicate and 4 blank wells. The assay lower limit of quantitation (LLOQ) (representing the lowest IgG titres that can be reliably and precisely quantified within a coefficient of variation of 25%) was determined mathematically. This was based on the 4-PL function of the standard curve data from 250 independent experiments and represents the EU value corresponding to the upper 95% CI of the minimum asymptote of the 4-PL curve fit used for modeling the assay standard curves. The value of 13 EU was calculated as the assay LLOQ, and this corresponds to an OD value of 0.2, for which the assay was demonstrated to show linearity.

### FRNT.

Antibody neutralization was measured in a randomly selected subset of participants using a FRNT, as described previously (69), where the reduction in the number of the infected foci is compared with a no-antibody negative control well. Briefly, serially diluted Ab or plasma was mixed with SARS-CoV-2 strain Victoria and incubated for 1 hour at 37°C. The mixtures were then transferred to 96-well, cell culture–treated, flat-bottom microplate containing confluent Vero cell monolayers in duplicate and incubated for further 2 hours, followed by the addition of 1.5% semisolid carboxymethyl cellulose (CMC) overlay medium to each well to limit virus diffusion. A focus-forming assay was then performed by staining Vero cells with human anti–NP mAb (mAb206) (69) followed by peroxidase-conjugated goat anti–human IgG (A0170, MilliporeSigma). Finally, the foci (infected cells) — approximately 100 per well in the absence of antibodies — were visualized by adding TrueBlue Peroxidase Substrate. Virus-infected cell foci were counted on the classic AID ELISpot reader using AID ELISpot software. The percentage of focus reduction was calculated and IC_50_ (reported as FRNT_50_) was determined using the probit program from the SPSS package. 

### MSD ACE-2 inhibition assay.

A multiplexed MSD immunoassay (MSD) was used to measure the ability of human sera to inhibit ACE-2 binding to SARS-CoV-2 spike (B, B.1 [D614G], B.1.1.7 [Alpha], B.1.351 [Beta], or P.1 [Gamma]). A MULTI-SPOT 96-well, 10-spot plate (plate 7) was coated with 8 SARS-CoV-2 spike and RBD antigens (B, B.1, B.1.1.7, B.1.351, or P.1). Multiplex MSD assays were performed as per manufacturer’s instructions. To measure ACE-2 inhibition, 96-well plates were blocked with MSD blocker for 30 minutes. Plates were then washed in MSD washing buffer, and samples were diluted 1:10 and 1:100 in diluent buffer. Importantly, an ACE-2 calibration curve, which consists of a monoclonal antibody with equivalent activity against spike variants, was used to interpolate results as AUs. Furthermore, internal controls and the WHO international standard were added to each plate. After 1-hour incubation, recombinant human ACE-2-SULFO-TAG was added to all wells. After a further 1 hour, plates were washed and MSD GOLD Read Buffer B was added; plates were then immediately read using a MESO SECTOR S 600 Reader.

### Isolation of peripheral blood mononuclear cells (PBMC) from whole blood.

PBMCs were isolated bydensity gradient centrifugation using Lymphoprep (Stem Cell Technologies). Buffy coats containing PBMCs were collected and washed twice with prewarmed R10 medium: RPMI 1640 medium (Sigma-Aldrich) supplemented with 10% heat-inactivated fetal calf serum (FCS; MilliporeSigma), 1 mM penicillin-streptomycin solution (MilliporeSigma), and 2 mM L-glutamine solution (MilliporeSigma). After the second centrifugation (300*g*, 7 minutes, room temperature), cells were resuspended in R10 and counted using the Guava ViaCount assay (Guava Technologies Hayward) on the Muse Cell Analyzer (Luminex Cooperation). T cell ELISpot assays were done on freshly isolated PBMCs, and CellTrace Violet (CTV; Thermo Fisher Scientific) T cell proliferation assays were done on cryopreserved samples.

### Ex vivo IFN-γ ELISpot to enumerate antigen-specific T cells.

ELISpot assays were performed as described previously (17) using a validated protocol with freshly isolated PBMCs to determine responses to the SARS-CoV-2 spike vaccine antigen at days 0 (before vaccination), 14, 28 (boost), 42, and 56. Assays were performed using Multiscreen IP ELISpot plates (Merck Millipore) coated with 10 μg/mL human anti–IFN-γ antibody and developed using SA-ALP antibody conjugate kits (Mabtech) and BCIP NBT-plus chromogenic substrate (Moss Inc.). PBMC were separated from whole blood with lithium heparin by density centrifugation within 4 hours of venepuncture. Cells were incubated 18–20 hours in RPMI (MilliporeSigma) containing 1000 units/mL penicillin, 1 mg/mL streptomycin, and 10% heat-inactivated, sterile-filtered fetal calf serum, previously screened for low reactivity (Labtech International) with a final concentration of 10μg/mL of each peptide. A total of 253 synthetic peptides (15 mers overlapping by 10 amino acids) spanning the entire vaccine insert, including the tissue plasminogen activator (tPA) leader sequence, were used to stimulate PBMC (ProImmune). Peptides were pooled into 12 pools for the SARS-CoV-2 spike protein containing 18–24 peptides, plus a single pool of 5 peptides for the tPA leader. Peptides were tested in triplicate, with 2.5 × 10^5^ PBMC added to each well of the ELISpot plate in a final volume of 100μL. Results are expressed as spot-forming cells (SFC) per million PBMCs, calculated by subtracting the mean negative control response from the mean of each peptide pool response and then summing the response for the 12 peptide pools spanning S1 and S2. Staphylococcal enterotoxin B (0.02 μg/mL) and phytohaemagglutinin-L (10 μg/ mL) were pooled and used as a positive control. Plates were counted using an AID automated ELISpot counter (AID Diagnostika GmbH, algorithm C) using identical settings for all plates, and counts were adjusted only to remove artefacts. A lower limit of detection of 48 SFC/million PBMCs was determined based on the minimum number of spots that could be detected.

### T cell proliferation assay.

T cell proliferation assay was done using cryopreserved PBMCs. Briefly, PBMCs were thawed and washed twice with 1 mL of PBS, followed by labelling with CTV at a final concentration of 2.5 μM for 10 minutes at RT. CTV, a free amine binding dye, enables the measurement of the decrease in dye concentration following each cell division in proliferating cells in response to antigenic stimulation, as described previously (33). The labeling reaction was quenched with 4 mL of FBS at 4°C, and cells were resuspended in RPMI medium supplemented with 10% human blood group type AB serum (MilliporeSigma), 1 mM penicillin-streptomycin solution, and 2 mM L-glutamine solution and were subsequently plated in a 96-well round-bottom plate at a plating density of 0.25 × 10^6^ cells per well in duplicate wells (total of 0.5 × 10^6^ cells per condition). Cells were stimulated with peptide pools (15 mers overlapping by 11) spanning SARS-CoV-2 spike (S1 and S2), SARS-CoV-2 VOCs (Beta, Gamma, and Delta) and HCoVs (HKU-1; 2 consensus clades, OC43 and NL63; and 299E) at a final concentration of 1 μg/mL per peptide. For antigenic control, class 1 and 2 optimal peptides for FEC-T (flu, EBV, CMV, and tetanus) were pooled at a final concentration of 1 μg/mL per peptide. Media, containing 0.1% DMSO (MilliporeSigma) representing DMSO content in peptide pools, was used as a negative control, and 2 μg/mL phytohaemagglutinin-L (MilliporeSigma) was used as positive control. Cells were then incubated at 37°C, with 5% CO_2_ and 95% humidity for 7 days, with a change of media on day 4. At the end of the incubation period, cells were stained using anti–human CD3, CD4, CD8, and a live cell discriminator (Live/Dead near Infra-red, Invitrogen, L34976) as in Supplemental Table 5. All samples were acquired using a BD Fortessa X20 (BD Biosciences) or MACSQuant x10 (Miltenyi Biotec). Responses above 1% were considered true positive based mean of DMSO controls + 3 SD. Specificity of the assay has been previous reported (33). All data points presented represent a single participant and are presented as background subtracted data.

### AIM assay.

Cryopreserved PBMCs from 25 HIV-infected subjects were used for AIM assay. Briefly, PBMCs were thawed in R10 (RPMI + 10% FCS, 1% penicillin-streptomycin, and 1% L-glutamine). Cells were washed, counted, and rested for 6 hours in IMDM-10 (IMDM, MilliporeSigma; I3390 + 10% human AB serum, 1% penicillin-streptomycin, and 1% L-glutamine) and 1 μL/mL of benzonase nucleases (70746-3, Merck). Following rest, cells were plated at 1 × 10^6^ to 2 × 10^6^ cells/well in a 96-well round-bottom plate. Cells were then incubated for 24 hours at 37°C and 5% CO_2_. After stimulation, cells were stained with the anti-human antibodies, which are detailed in Supplemental Table 6. Stained cells were fixed in 4% PFA and acquired on a BD LSR II flow cytometer. The data were analyzed using FlowJo version 10 and Prism version 9. Antigen-specific CD4^+^ and CD8^+^ T cells were gated using the Boolean OR gating strategy described by Nielsen et al. (47) and shown in Supplemental Figure 5 (for CD4 T cells, all double-positive CD25^+^ CD134(OX40)^+^, CD25^+^CD137^+^, or CD25^+^CD69^+^ were considered AIM^+^; for CD8^+^ T cells, all double-positive CD25^+^CD137^+^ or CD25^+^CD69^+^ were considered AIM^+^). Chemokine receptors CCR6 and CXCR3 were used as an unbiased way of analyzing T cell skewness independently of cytokine kinetics.

### Ex vivo phenotyping, activation, and exhaustion assays.

Cryopreserved PBMCs were thawed in 30 mL of RPMI media supplemented with 10% FBS, 1% penicillin-streptomycin, and 1% L-glutamine (R10). Cells were counted and rested for an hour at a cell density of 2 × 10^6^ per mL of R10 in the presence of benzonase endonucleases(70746-3, Merck). Following rest, 2 million to 3million cells wereused for each of the panels. Cells were washed in staining buffer (420201, BioLegend). This was followed by blocking FC receptors (422302, BioLegend) for 10 minutes at RT and live cell staining using L/D aqua (L34966, Invitrogen). All cells were then washed in preparation for antibody staining. For ex vivo immuneactivation panel, antibodies for assessing immune activation (as listed in Supplemental Table 7 with manufacturer names, catalog numbers, and dilution) were used as a cocktail and added to the cell pellet. Cells were subsequently incubated at 37°C for 15 minutes, which was followed by a wash and fixation in 4% paraformaldehyde (PFA) for 10 minutes at RT. PFA was washed off and cells were resuspended in PBS for acquisition on flow cytometer. For immune exhaustion panel, antibodies for surface markers were prepared in a cocktail that was added to cells and incubated for 15 minutes at 37°C (Supplemental Table 8). Cells were then prepared for intranuclear stain using FOXP3 fixation/permeabilization kit (Invitrogen). Briefly, 100 μL of fixation buffer was added to cells and incubated at RT for 30 minutes. This was followed by cellular permeabilization using the permeabilization buffer contained in the aforementioned kit. Antibody cocktails were prepared in permeabilization buffer, added to cells, and allowed to incubate for 30 minutes at RT. Following staining, cells were washed and resuspended in PBS for acquisition. All data were acquired on a BD LSR II flow cytometer, and fluorescence minus 1 (FMO) gates were used to set gates for markers of interest. Gating strategies are as shown in Supplemental Figure 1, A and B.

### Phylogenetic analysis.

We used protein BLAST to download all human coronavirus S protein sequences from the NCBI database (https://www.ncbi.nlm.nih.gov/sars-cov-2/). We then randomly chose 3 sequences for each of the human coronavirus species. HKU1 consisted of 2 clades, and we chose 3 isolates for each clade (c1 and c2). We used MAFFT to align all chosen human coronaviruses, SARS-CoV, MERS-CoV, and SARS-CoV-2 S protein sequences. We then calculated the pairwise distances between the sequences and built a neighbor-joining tree using MATLAB.

### Statistics.

This study was not powered to a specific endpoint, and the sample size was based on practical recruitment considerations in line with other subgroups of the COV002 study. We analyzed all outcomes in all participants who received both doses of the vaccination schedule and with available samples, unless otherwise specified. We log-transformed serological, FRNT_50_, and ELISpot data for analysis. FRNT_50_ titres less than 20 were given the value 10 for statistical analysis. We present medians and IQRs for immunological endpoints. We used nonparametric analysis (Spearman’s ρ) for correlations between 2 immunological endpoints. For comparison of 2 nonparametrically distributed unpaired variables, we used the Wilcoxon rank sum (Mann Whitney *U*) test. For comparison of 2 nonparametrically distributed paired data sets, we used the Wilcoxon matched-pairs signed-rank test. We used the χ^2^ test for comparison of ELISpot responses. Missing data were not inputed. We did all analyses using R (version 3.6.1 or later) and Prism 9 (GraphPad Software), and *P* < 0.05 was considered statistically significant.

### Study approval.

Written informed consent was obtained from all participants prior to participation, and the trial was done in accordance with the principles of the Declaration of Helsinki and Good Clinical Practice. Study approval in the United Kingdom was done by the Medicines and Healthcare products Regulatory Agency (no. 21584/0424/001-0001) and the South Central Berkshire Research Ethics Committee (no. 20/SC/0145). Vaccine use was authorized by Genetically Modified Organisms Safety Committees at each participating site. The COV002 study is registered with ClinicalTrials.gov (NCT04400838) and is ongoing.

## Author contributions

J Frater, AO, SF, J Fox, TL, AJP, SCG, AVSH, and KJE were involved in conceptualization, data curation, funding acquisition, supervision, methodology, and writing and reviewing the manuscript. AO, MP, SA, MAA, A Brown, HB, MJ, LP, NR, T Tipoe, and PZ were involved with sample methodology and preparation, assay performance, data curation (CTV assays), data analysis, and writing and reviewing the manuscript. EB, SD, PG, and PK were involved in supervision, data curation, investigation, methodology, and writing and reviewing the manuscript. SL, T Tipton, and MC oversaw MSD and ACE-inhibition assays. JA, A Bara, CP, KMP, HS, AW, and SF managed study recruitment at Imperial. PC, AM, TR, BJ, MM, SB, ALG, SS, AW, and J Fox managed study recruitment at Guy’s and St Thomas’ NHS Trust; WD and GRS were involved in data curation (neutralization), formal analysis, methodology, and writing and reviewing the manuscript. AB, JA, CP, KMP, HS, AW, and SF were involved in data curation, methodology, project administration, supervision, investigation, and writing and reviewing the manuscript. PKA was involved in project administration. MB, FC, PMF, J Fowler, LG, DJ, RAM, and TL were involved in data curation, formal analysis, investigation, methodology, and writing and reviewing the manuscript. SB, HF, ALG, SS, AW, and J Fox were involved in data curation, methodology, project administration, supervision, investigation, and writing and reviewing the manuscript. NGM, YFM, EP, MNR, SR, HR, and AJP were involved in data curation, formal analysis, investigation, methodology, and writing and reviewing the manuscript. All authors critically reviewed and approved the final version. Co–first authorship was assigned based on relative contribution to the study. Order of first authors was agreed between them and reflect contribtions to the work undertaken. AO led the writing of the manuscript.

## Supplementary Material

Supplemental data

## Figures and Tables

**Table 1:**
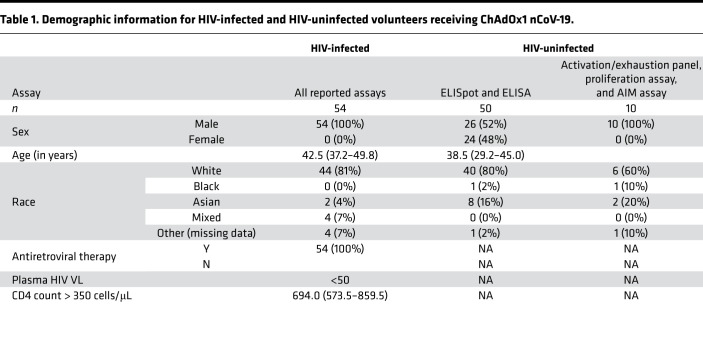
Demographic information for HIV-infected and HIV-uninfected volunteers receiving ChAdOx1 nCoV-19.

## Version 1 02/22/2022

In-Press Preview

## Version 2 04/08/2022

Electronic publication
