# Supplementary material for: Durability of ChAdOx1 nCoV-19 vaccination in people living with HIV
Source: JCI Insight. 2022 Apr 8;7(7):e157031. doi: 10.1172/jci.insight.157031 (PMC9057612; doi:10.1172/jci.insight.157031)
Supplement: Supplemental data [file jciinsight-7-157031-s216.pdf]

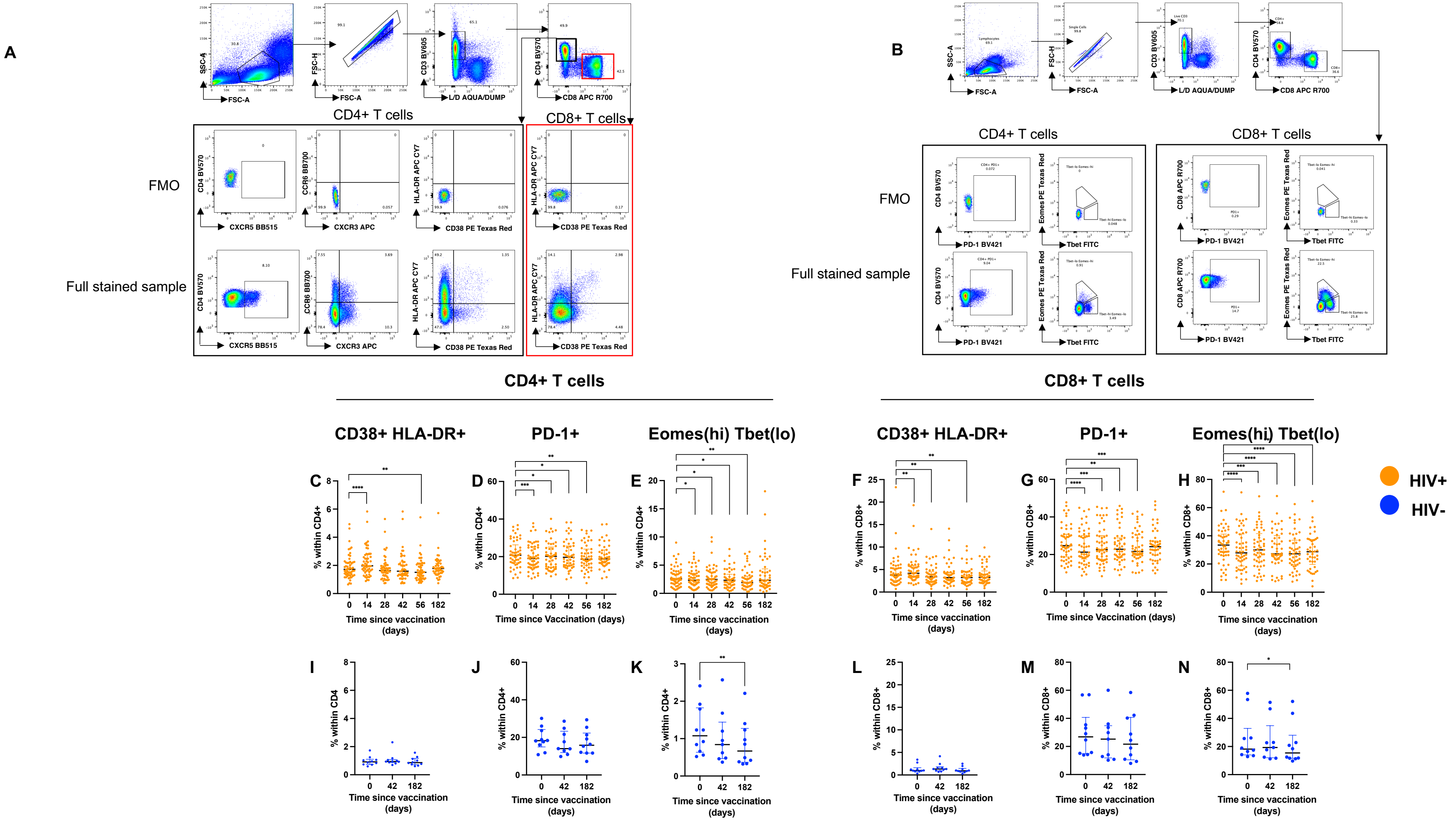

Supplementary figure 1: Flow cytometry gating strategy and kinetics of cells expressing activation and exhaustion marker.
Gating strategy for **(A)** activation panel and **(B)** exhaustion panel. All gates were set based on fluorescent minus one (FMO). Frequency of cells expressing **(C)** CD38+ HLA DR+, **(D)** PD-1+, **(E)** Eomes(hi) Tbet(lo) within CD4+ T cells on HIV+ participants and frequency of cells expressing **(F)** CD38+ HLA DR+, **(G)** PD-1+, **(H)** Eomes(hi) Tbet(lo) within CD8+ T cells on HIV+ participants. Frequency of cells expressing **(I)** CD38+ HLA DR+, **(J)** PD-1+, **(K)** Eomes(hi) Tbet(lo) within CD4+ T cells on HIV- participants and frequency of cells expressing **(L)** CD38+ HLA DR+, **(M)** PD-1+, **(N)** Eomes(hi) Tbet(lo) within CD8+ T cells on HIV- participants. Comparison of two timepoints within the same group was done by Wilcoxon matched pair sign ranked test. Where indicated * = <0.05, ** = <0.01, *** = < 0.001 and **** = <0.0001. n = 48 – 54 for HIV+ volunteers and 10 for HIV- controls. Error bars represent median and interquartile range.

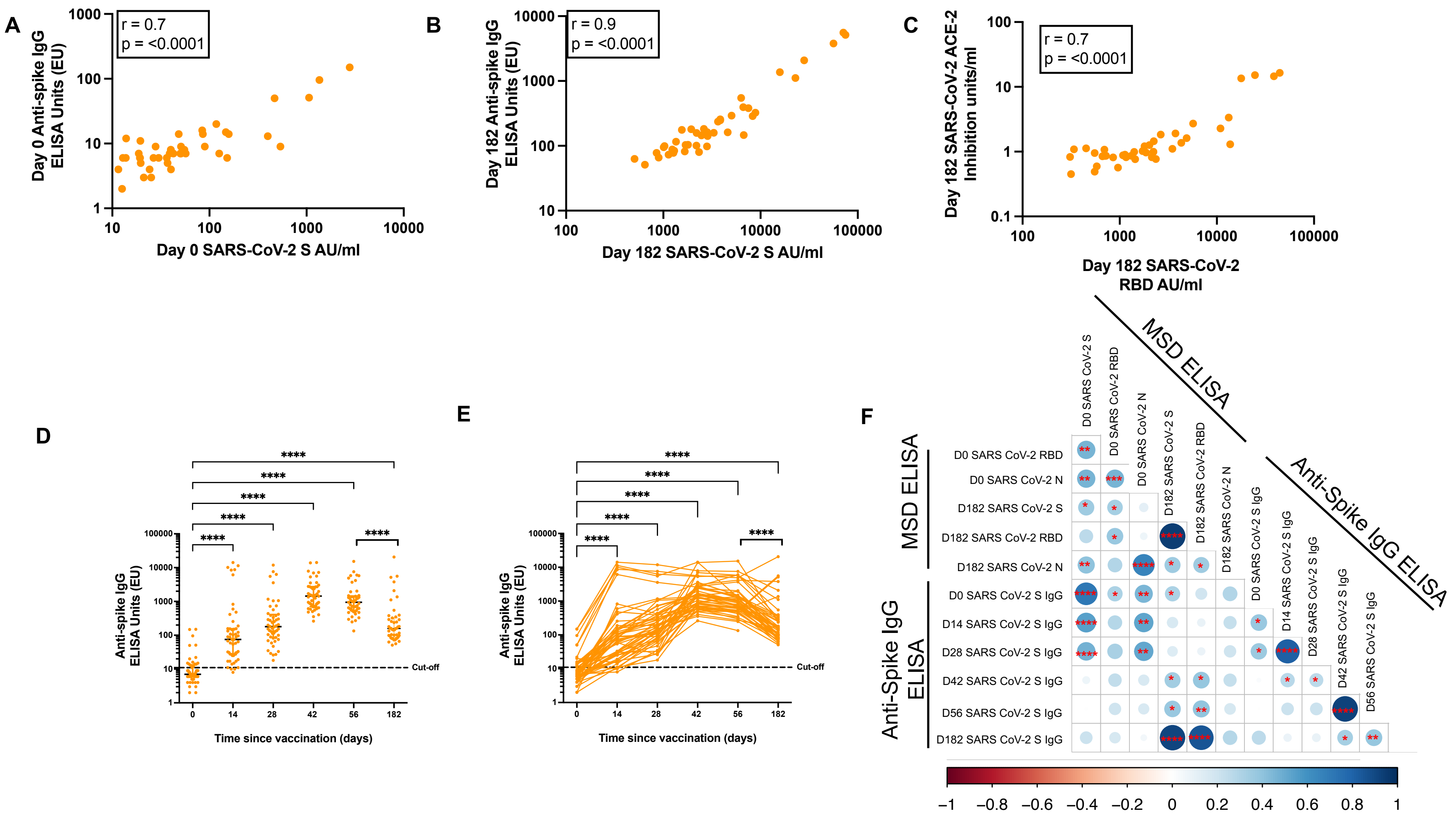

Supplementary figure 2: Humoral immune responses against SARS-CoV-2 in PWH.
(A) Correlations between antibody levels measured using MSD assay and in-house total IgG ELISA at **(A)** day 0, **(B)** day 182 and **(C)** correlations between day 182 SARS-CoV-2 RBD levels and ACE-2 binding inhibition assay. **(D)** Antibody levels in HIV+ participants measured across all timepoints presented as dot plots and, **(E)** before-after plots to show individual responses. **(F)** Correlation plot showing correlation matrix between SARS-CoV-2 humoral immune response across all proteins, assays and timepoints. Correlation was performed via Spearman's rank correlation coefficient and correlation matrix was created using corrplot package on R studio. Size and Colour of the heatmap corresponds to the correlation coefficient. Comparison of two timepoints within the same group was done by Wilcoxon matched pair sign ranked test. Where indicated * = <0.05, ** = <0.01, *** = < 0.001 and **** = <0.0001. n = 42 – 54 for HIV+ volunteers. Error bars represent median and interquartile range.

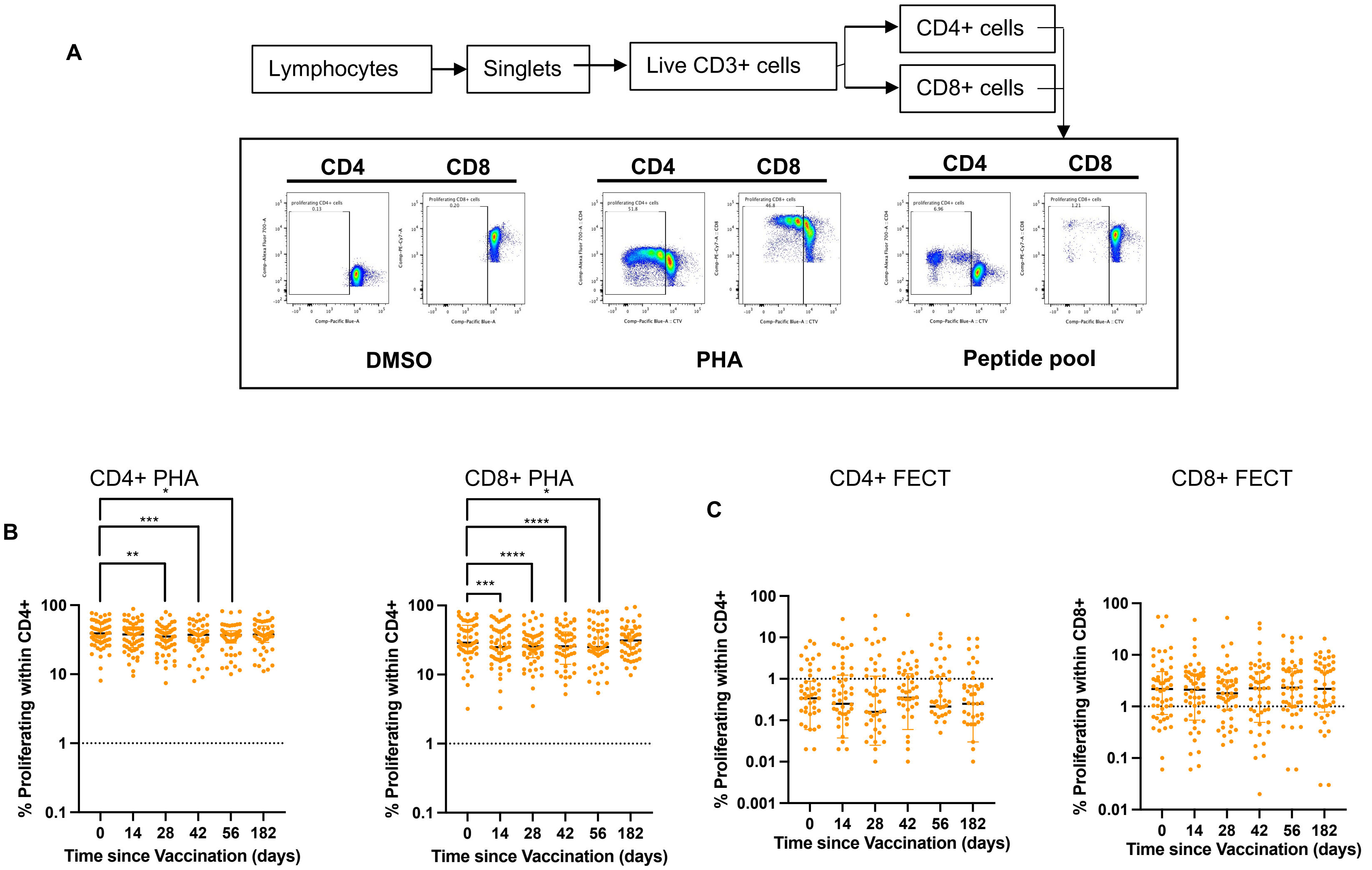

Supplementary figure 3: Gating strategy and T cell response to control antigens (FECT) and mitogen (PHA).
(A) Gating strategy for proliferation assay. All gates were set based on DMSO controls. Background was subtracted and responses were assigned positive if they were >1% after background subtraction. **(B)** PHA responses in CD4+ and CD8+ T cells at longitudinal timepoints. **(C)** FECT responses in CD4+ and CD8+ T cells at longitudinal timepoints. Comparison of two timepoints within the same group was done by Wilcoxon matched pair sign ranked test. Where indicated * = <0.05, ** = <0.01, *** = < 0.001 and **** = <0.000. Dotted lines in indicate threshold for true positive based mean of DMSO controls + 3x SD. n = 48 – 54 for HIV+ volunteers. Error bars represent median and interquartile range.

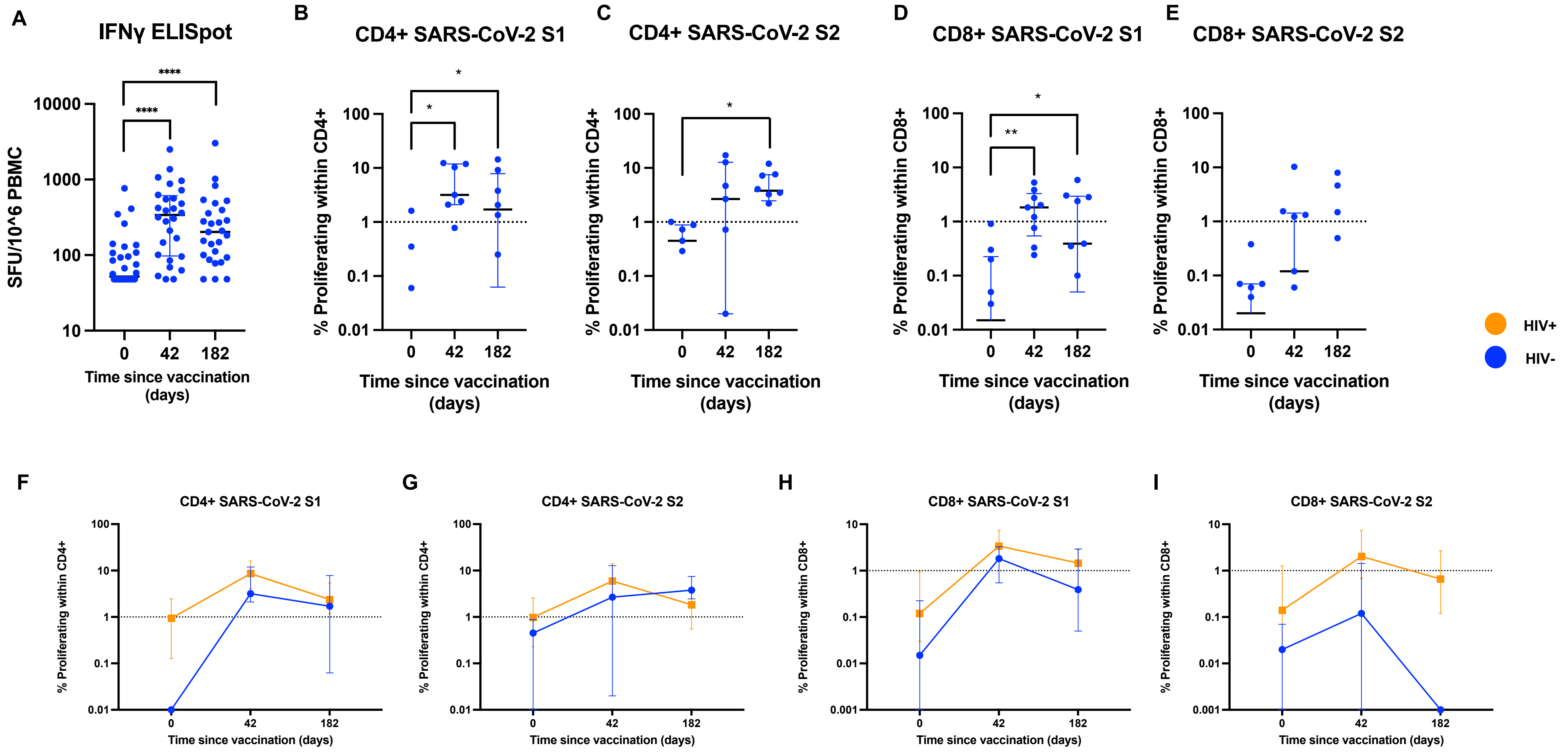

Supplementary figure 4: Longitudinal T cell responses to SARS-CoV-2 in HIV+ and HIV- subjects following ChAdOx1 nCoV-19 vaccination. **(A)** IFNγ ELISpot responses in HIV- volunteers at day 0, 42 and 182. T cell proliferative response to **(B)** SARS-CoV-2 S1, **(C)** SARS-CoV-2 S2 in CD4+ T cells in HIV- volunteers and T cell proliferative response to **(D)** SARS-CoV-2 S1, **(E)** SARS-CoV-2 S2 in CD8+ T cells in HIV- volunteers. Comparison of T cell proliferative responses to **(F)** SARS-CoV-2 S1, **(G)** SARS-CoV-2 S2 in CD4+ T cells **(H)** SARS-CoV-2 S1, **(I)** SARS-CoV-2 S2 in CD8+ T cells in HIV+ and HIV negative volunteers at day 0, 42 and 182. Comparison of two timepoints within the same group was done by Wilcoxon matched pair sign ranked test. Comparison of two groups was done by two-tailed multiple Mann-Whitney U test Bonferonni-Dunn's multiple comparison test (Prism v9). Where indicated * = <0.05, ** = <0.01, *** = < 0.001 and **** = <0.000. Dotted lines in indicate threshold for true positive based mean of DMSO controls + 3x SD. n = 48 – 54 for HIV+ volunteers, 54 for HIV- control ELISPOTS, 10 for HIV- control proliferation assay. Error bars represent median and interquartile range.

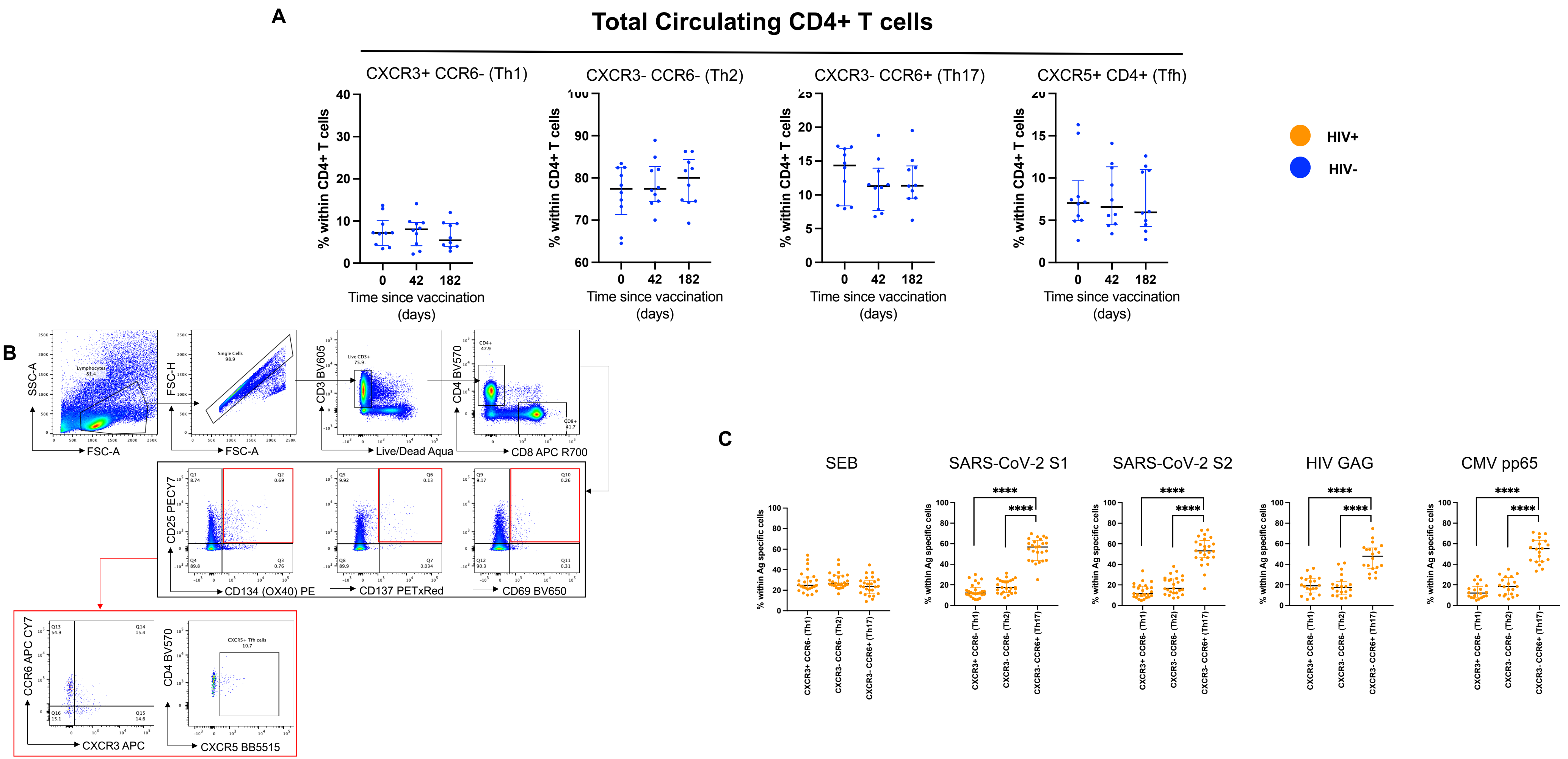

Supplementary figure 5: Phenotype of total and SARS-CoV-2 S-specific circulating CD4+ T cells

(A) *Ex vivo* frequencies of **(A)** CXCR3+ CCR6+ (Th1), CXCR3- CCR6- (Th2), CXCR3- CCR6+ (Th17), and CXCR5+ within CD4+ T cells in HIV- volunteers measured at various timepoints. **(B)** gating strategy for AIM assay. All cells expressing CD25+ CD134 (OX40) and CD25+ CD137+ and CD25+ CD69+ were Boolean gated as antigen specific cells. Gating was set based on DMSO control and all responses were background subtracted. CXCR3 and CCR6 quadrant gate for antigen specific population was set based on expression in bulk CD4 T cell population. **(C)** Frequency of CD4 T cell subsets in SEB, SARS-CoV-2 S1, SARS-CoV-2 S2, HIV GAG and CMVpp65. Comparison of two timepoints within the same group was done by Wilcoxon matched pair sign ranked test. Comparison of two groups was done by two-tailed multiple Mann-Whitney U test. Where indicated * = <0.05, ** = <0.01, *** = < 0.001 and **** = <0.000. n = 18 – 20 for HIV+ volunteers and 10 for HIV- controls. Error bars represent median and interquartile range.

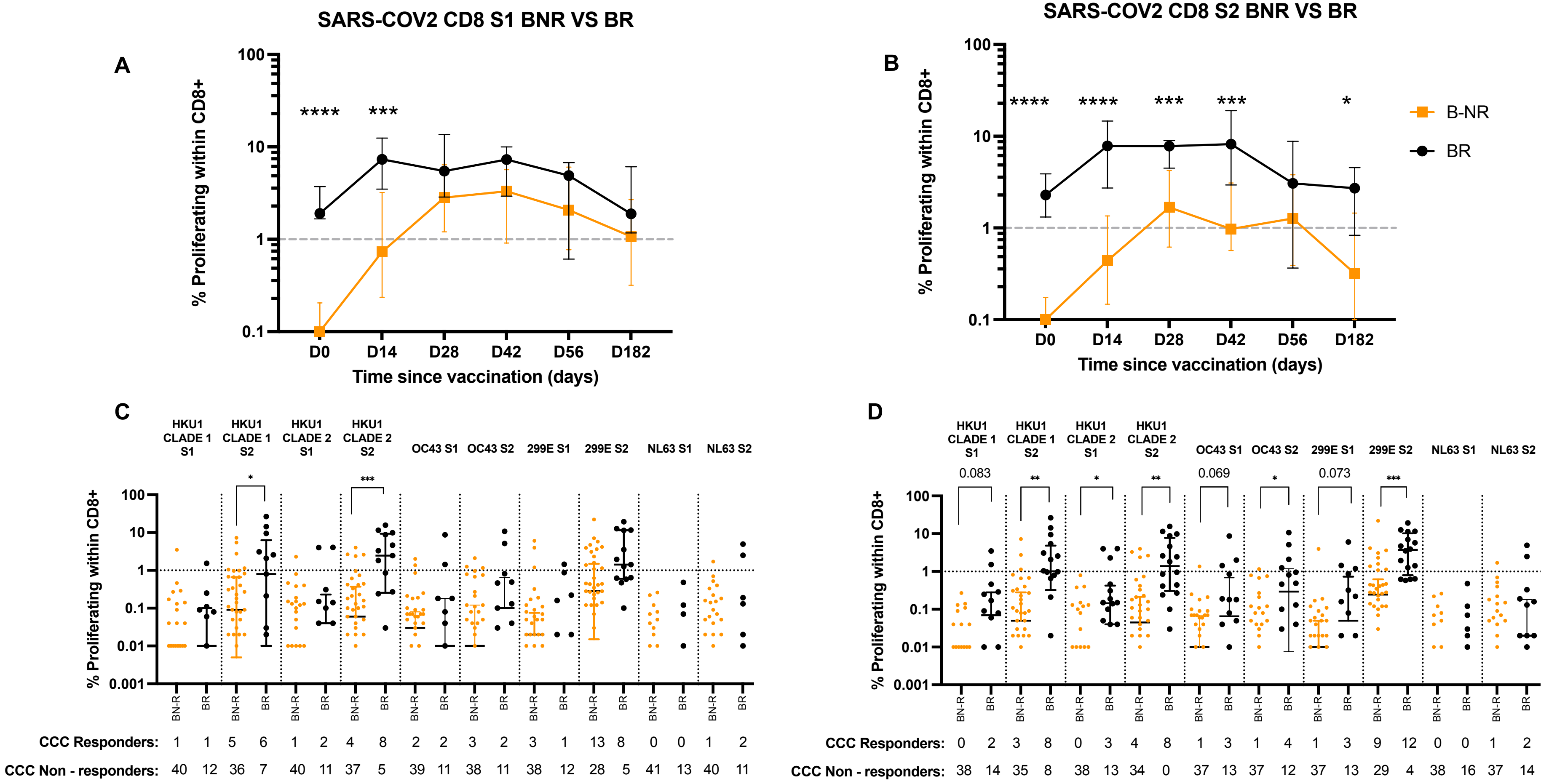

Supplementary figure 6: Pre-existing cross-reactive immunity in PWH measured at baseline are associated with high magnitude CD8+ T cell responses post ChAdOx1 nCoV-19 vaccination

Baseline CD8+ SARS-CoV-2 responses were split into baseline responders (BR, proliferation >1%, black circles and black lines) and baseline non-responders (B-NR, Proliferation <1%, yellow circles and yellow lines) and CD8 T cell responses post vaccination were analysed at all available timepoints for **(A)** SARS-CoV-2 S1 and **(B)** SARS-CoV-2 S2. T cells responses targeting **(C)** S1 and **(D)** S2 proteins in endemic CCCs are measured at baseline in BR and B-NR. Comparison of two timepoints within the same group was done by Wilcoxon matched pair sign ranked test. Comparison of two groups was done by two-tailed multiple Mann-Whitney U test with Bonferonni-Dunn's multiple comparison test (Prism v9). **A** and **B** show adjusted significant levels. CCC responses among participants were compared using fisher's exact test and listed in supplementary table 3. P values as indicated or * = <0.05, ** = <0.01, *** = < 0.001 and **** = <0.000. Dotted lines in indicate threshold for true positive based mean of DMSO controls + 3x SD. n = 48 – 54 for HIV+ volunteers. Error bars represent median and interquartile range.

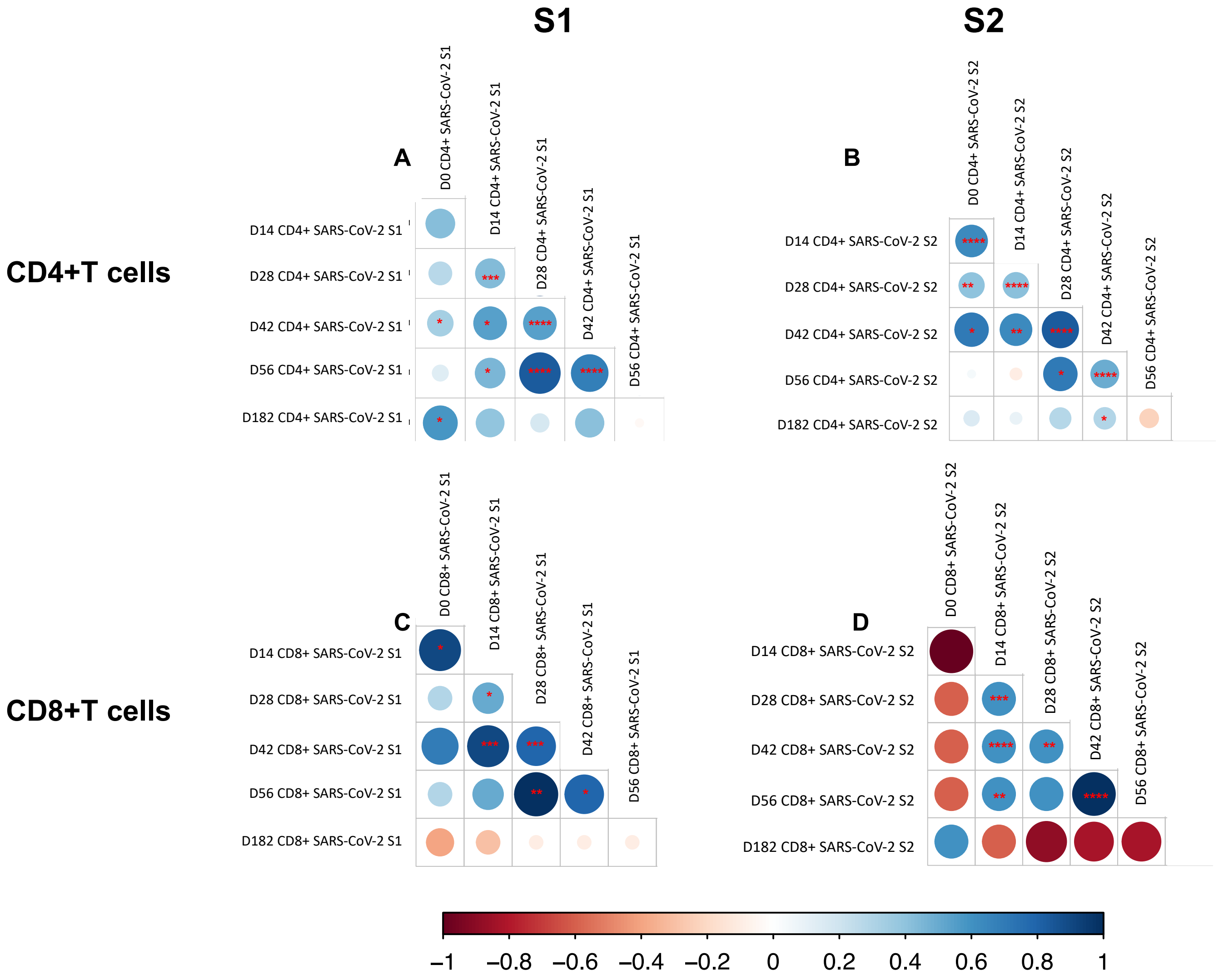

Supplementary figure 7: Relationship between baseline and post vaccination. timepoints for CD4+ and CD8+ proliferative T cells. Correlation plots showing correlation matrix for **(A)** CD4+ SARS-CoV-2 S1, **(B)** CD4+ SARS-CoV-2 S2, **(C)** CD8+ SARS-CoV-2 S1, **(D)** CD8+ SARS-CoV-2 S2. Correlation was performed via Spearman's rank correlation coefficient and correlation matrix was created using corrplot package on R studio. Size and Colour of the heatmap corresponds to the correlation coefficient. Where indicated * = <0.05, ** = <0.01, *** = < 0.001 and **** = <0.0001. n = 42 – 54 for HIV+ volunteers.

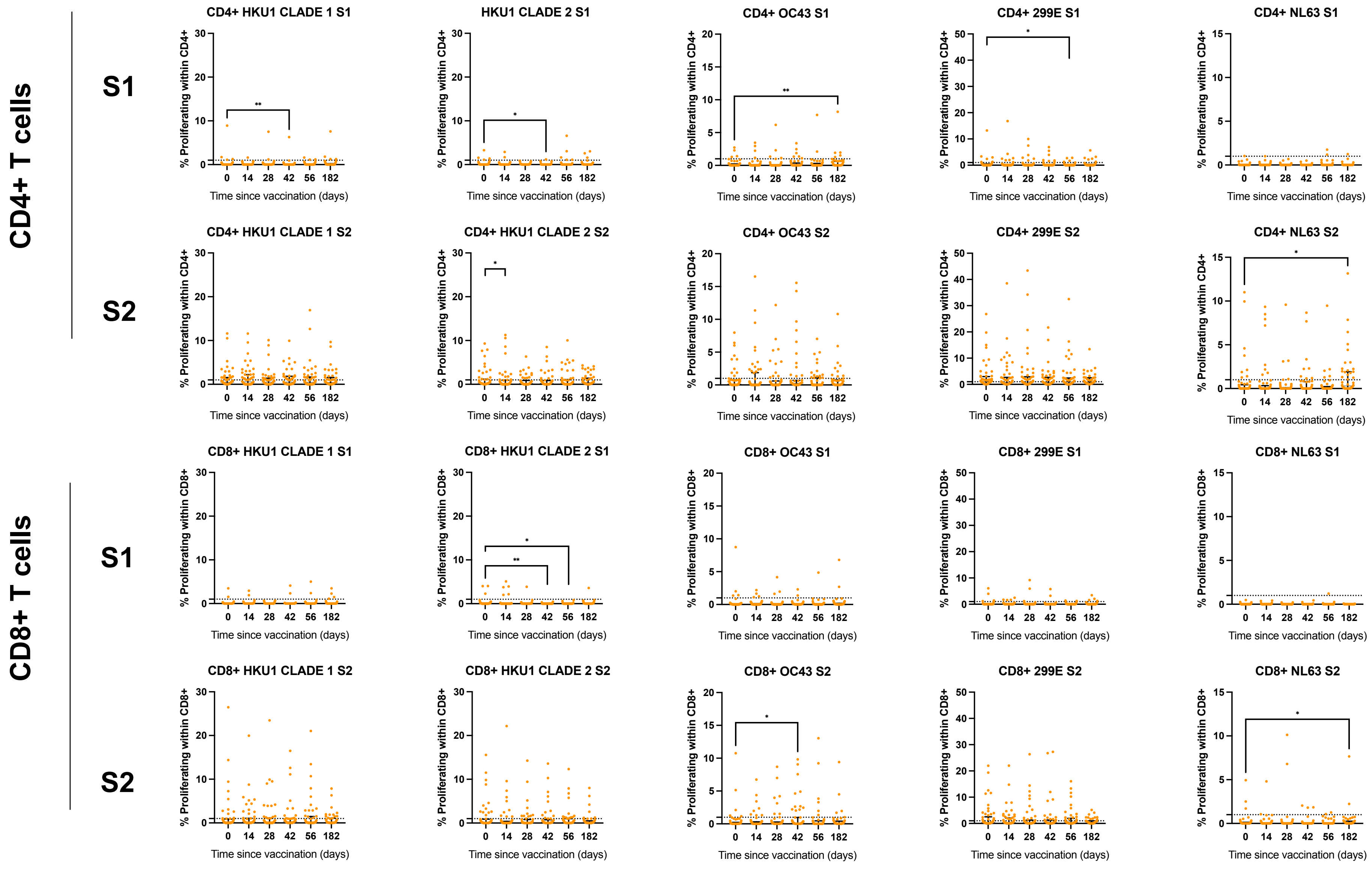

Supplementary figure 8: Responses to CCC in PWH

T cell proliferative responses to CCCs HKU-1 clade 1 and 2, OC43, 299E, NL63 S1 and S2 in CD4+ and CD8+ T cells measured on day 0, 14, 28, 42, 56 and 182. Comparison of two timepoints within the same group was done by Wilcoxon matched pair sign ranked test. P values as indicated or * = <0.05, ** = <0.01, *** = < 0.001 and **** = <0.000. Dotted lines in indicate threshold for true positive based mean of DMSO controls + 3x SD. n = 46 – 54 for HIV+ volunteers. Error bars represent median and interquartile range.

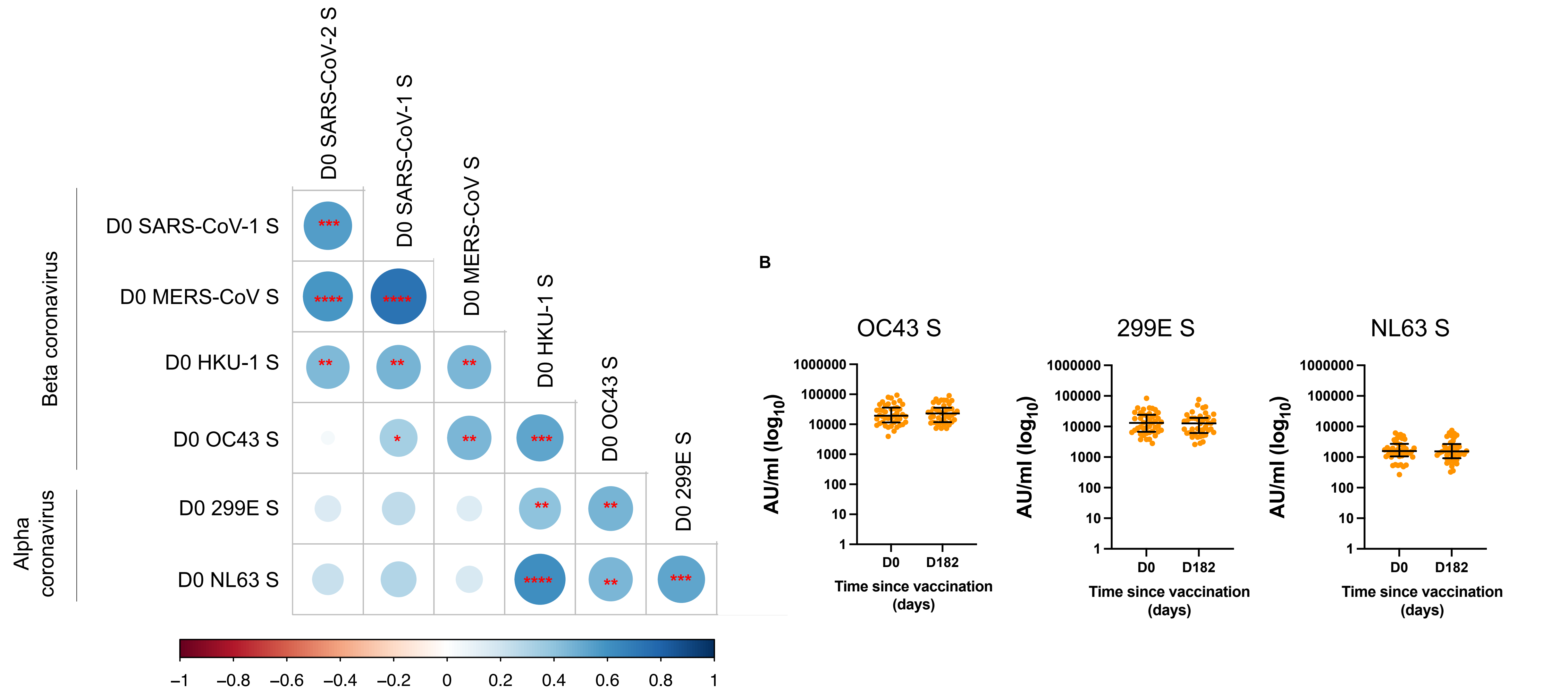

Supplementary figure 9: Relationship between antibody responses for SARS-CoV-2 and CCCs spike in PWH

(A) Correlation plot showing correlation matrix between different circulating HCoVs. **(B)** Antibody titres at day 0 and day 182 for OC43 Spike, 299E spike and NL63 spike proteins in HIV+ participants. Correlation was performed via Spearman's rank correlation coefficient and correlation matrix was created using corrplot package on R studio. Size and Colour of the heatmap corresponds to the correlation coefficient. Comparison of two timepoints within the same group was done by Wilcoxon matched pair sign ranked test. Where indicated * = <0.05, ** = <0.01, *** = < 0.001 and **** = <0.0001. n = 42 – 54 for HIV+ volunteers. Error bars represent median and interquartile range.

Supplementary table 1: T cell proliferation panel using Cell trace violet® (CTV) cell dye

	Fluorochrome	Marker	Clone	Volume/50ul	Catalogue No	Company
CTV Proliferation Assay		L/D NIR			L24976	Invitrogen
		CTV			C34557	Invitrogen
	FITC	CD3	UCHT1	1	300440	Biolegend
	APC	CD4	RPA-T4	0.25	300537	Biolegend
	PE CY7	CD8	RPA-T8	0.25	301012	Biolegend

Supplementary table 2: Ex vivo activation panel

	Fluorochrome	Marker	Clone	Volume/50ul	Catalogue No	Company
Ex vivo Activation Panel	L/D AQUA	Viability discriminator		1		
	BV510	CD14	M5E2	0.25	302242	Biolegend
	BV510	CD19	HIB19	0.25	301842	Biolegend
	BV6711	CD3	UCHT1	1	300464	Biolegend
	BV570	CD4	RPA-T4	1	300534	Biolegend
	BB515	CXCR5	RF8B2	1	564624	BD Bioscience
	APC CY7	HLA-DR	L243	1.25	307617	Biolegend
	BB700	CCR6	11A9	1	566478	BD Bioscience
	APCR700	CD8	RPA-T8	1	565165	BD Bioscience
	PE TxR	CD38	HIT2	0.5	562288	BD Bioscience
	APC	CXCR3	IC6/CXCR3	10	550967	BD Bioscience

Supplementary table 3: Ex vivo exhaustion panel

	Fluorochrome	Marker	Clone	Volume/50ul	Catalogue No	Company
T cell exhaustion Panel	L/D AQUA	Viability discriminator				
	BV510	CD14	M5E2	0.25	302242	Biolegend
	BV510	CD19	HIB19	0.25	301842	Biolegend
	BV605	CD3	UCHT1	0.5	300460	Biolegend
	BV570	CD4	RPA-T4	1	300534	Biolegend
	BV421	PD1	EH12.2H7	1	329920	Biolegend
	APCR700	CD8	RPA-T8	0.5	565165	BD Bioscience
	PE TXR	EOMES	WD1928	1.25	61-4877-42	Invitrogen
	FITC	TBET	4B10	1.25	644812	Biolegend

Supplementary table 4: Activation induced marker (AIM) panel

	Fluorochrome	Marker	Clone	Volume/50ul	Catalogue No	Company
AIM assay	APC	CXCR3	1C6/CXCR3	5	550967	BD
	BB515	CXCR5	RF8B2	1	564624	BD
	APC CY7	CCR6	G034E3	2	353432	Biolegend
	BV421	PD1	EH12.2H7	1	329920	Biolegend
	BV510	CD19	HBI19	0.25	302242	Biolegend
	BV510	CD14	M5E2	0.25	301842	Biolegend
	PE CF 594	CD137	4B4-1	1	309826	Biolegend
	BV650	CD69	FN50	0.5	310934	Biolegend
	BV570	CD4	SK3	1	300534	Biolegend
	PERCP EFLUOR 710	CD39	eBioA1	1.5	46-0399-42	Invitrogen
	PE	CD134 (OX40)	L106	2	340420	BD
		L/D AQUA			L34966	Invitrogen
	PECY7	CD25	2A3	1	335824	BD
	APCR700	CD8	RPA-T8	0.25	565165	BD
	BV605	CD3	UCHT1	0.5	300460	Biolegend

Supplementary table 5: P values (by fisher test) for SARS-CoV-2 responders vs non-responders with CCC responses

		HKU1 clade 1 S1	HKU1 clade 1 S2	HKU1 clade 2 S1	HKU1 clade 2 S2	OC43 S1	OC43 S2	299E S1	299E S2	NL63 S1	NL63 S2
CD4+	SARS-CoV-2 S1	0.342	0.143	0.342	0.033	0.342	0.036	0.242	0.064	0.481	>0.999
	SARS-CoV-2 S2	0.111	>0.999	0.61	0.066	0.61	0.011	0.42	0.002	>0.999	>0.999
CD8+	SARS-CoV-2 S1	0.427	0.015	0.14	0.0004	0.242	0.584	>0.9999	0.109	>0.999	0.14
	SARS-CoV-2 S2	0.083	0.0012	0.022	0.003	0.069	0.023	0.073	0.0007	>0.999	0.206

Supplementary table 6: Spearman's correlations and P values for SARS-CoV-2 specific antibody responses measured using MSD technology and anti-spike IgG.

		MSD_D0_SARS-CoV-2 S	MSD_D0_SARS-CoV-2 RBD	MSD_D0_SARS-CoV-2 N	MSD_D182_SARS-CoV-2 S	MSD_D182_SARS-CoV-2 RBD	MSD_D182_SARS-CoV-2 N	anti-spike IgG D0_SARS-CoV2 S IgG	anti-spike IgG D14_SARS-CoV2 S IgG	anti-spike IgG D28_SARS-CoV2 S IgG	anti-spike IgG D42_SARS-CoV2 S IgG	anti-spike IgG D56_SARS-CoV2 S IgG	anti-spike IgG D182_SARS-CoV2 S IgG
Spearman's correlation	MSD_D0_SARS-CoV-2 S	1											
	MSD_D0_SARS-CoV-2 RBD	0.420433	1										
	MSD_D0_SARS-CoV-2 N	0.409726	0.490278	1									
	MSD_D182_SARS-CoV-2 S	0.357117	0.330848	0.112394	1								
	MSD_D182_SARS-CoV-2 RBD	0.262469	0.377846	0.071634	0.947978	1							
	MSD_D182_SARS-CoV-2 N	0.404068	0.292301	0.669679	0.352836	0.350729	1						
	anti-spike IgG D0_SARS-CoV2 S IgG	0.70605	0.313104	0.443993	0.317133	0.190068	0.296292	1					
	anti-spike IgG D14_SARS-CoV2 S IgG	0.564124	0.223708	0.451171	0.169889	0.090861	0.232805	0.369578	1				
	anti-spike IgG D28_SARS-CoV2 S IgG	0.495318	0.268454	0.457446	0.147413	0.085903	0.204482	0.359264	0.82714	1			
	anti-spike IgG D42_SARS-CoV2 S IgG	0.126326	0.188765	0.114547	0.34592	0.384329	0.234198	0.028872	0.32748	0.30319	1		
	anti-spike IgG D56_SARS-CoV2 S IgG	0.059277	0.153962	0.116061	0.376079	0.399092	0.178938	0.001634	0.265899	0.261139	0.928833	1	
	anti-spike IgG D182_SARS-CoV2 S IgG	0.253341	0.119228	0.011176	0.9122	0.856205	0.28583	0.261829	0.150604	0.153602	0.382301	0.420977	1
P value	MSD_D0_SARS-CoV-2 S	NA											
	MSD_D0_SARS-CoV-2 RBD	0.004999	NA										
	MSD_D0_SARS-CoV-2 N	0.006362	0.000846	NA									
	MSD_D182_SARS-CoV-2 S	0.02025	0.032346	0.478532	NA								
	MSD_D182_SARS-CoV-2 RBD	0.093104	0.013623	0.652125	0	NA							
	MSD_D182_SARS-CoV-2 N	0.007958	0.060324	1.24E-06	0.021912	0.02277	NA						
	anti-spike IgG D0_SARS-CoV2 S IgG	1.23E-07	0.040916	0.00286	0.040718	0.227958	0.056745	NA					
	anti-spike IgG D14_SARS-CoV2 S IgG	8.16E-05	0.149282	0.002393	0.282097	0.567144	0.137891	0.014723	NA				
	anti-spike IgG D28_SARS-CoV2 S IgG	0.000733	0.081753	0.002042	0.351535	0.588566	0.193952	0.01798	8.13E-12	NA			
	anti-spike IgG D42_SARS-CoV2 S IgG	0.419547	0.225408	0.464527	0.024836	0.011974	0.135484	0.854184	0.032061	0.048114	NA		
	anti-spike IgG D56_SARS-CoV2 S IgG	0.705742	0.32426	0.458603	0.014105	0.008841	0.256865	0.991705	0.084814	0.090754	0	NA	
	anti-spike IgG D182_SARS-CoV2 S IgG	0.101175	0.446349	0.943297	0	4.89E-13	0.066509	0.089873	0.335051	0.325406	0.011408	0.004937	NA

Supplementary table 7a: Spearman's correlations and P values for proliferative CD4+ T cell response to SARS-CoV-2 S1

		D0 CD4+ SARS-CoV-2 S1	D14 CD4+ SARS-CoV-2 S1	D28 CD4+ SARS-CoV-2 S1	D42 CD4+ SARS-CoV-2 S1	D56 CD4+ SARS-CoV-2 S1	D182 CD4+ SARS-CoV-2 S1
Spearman's correlation	D0 CD4+ SARS-CoV-2 S1	1					
	D14 CD4+ SARS-CoV-2 S1	0.37153846	1				
	D28 CD4+ SARS-CoV-2 S1	0.2756917	0.54032014	1			
	D42 CD4+ SARS-CoV-2 S1	0.4839074	0.36236934	0.59410277	1		
	D56 CD4+ SARS-CoV-2 S1	0.16753247	0.39080675	0.58717349	0.70557491	1	
	D182 CD4+ SARS-CoV-2 S1	0.5252838	0.20527859	0.16472895	0.2553094	0.24133033	1
P value	D0 CD4+ SARS-CoV-2 S1	NA					
	D14 CD4+ SARS-CoV-2 S1	0.06744806	NA				
	D28 CD4+ SARS-CoV-2 S1	0.20291642	0.00018398	NA			
	D42 CD4+ SARS-CoV-2 S1	0.02249512	0.01989648	2.67E-05	NA		
	D56 CD4+ SARS-CoV-2 S1	0.46791574	0.01265445	2.24E-05	2.58E-07	NA	
	D182 CD4+ SARS-CoV-2 S1	0.02518359	0.25970654	0.34433312	0.14503634	0.17606223	NA

Supplementary table 7b: Spearman's correlations and P values for proliferative CD4+ T cell response to SARS-CoV-2 S2

		D0 CD4+ SARS-CoV-2 S2	D14 CD4+ SARS-CoV-2 S2	D28 CD4+ SARS-CoV-2 S2	D42 CD4+ SARS-CoV-2 S2	D56 CD4+ SARS-CoV-2 S2	D182 CD4+ SARS-CoV-2 S2
Spearman's correlation	D0 CD4+ SARS-CoV-2 S2	1					
	D14 CD4+ SARS-CoV-2 S2	0.75395257	1				
	D28 CD4+ SARS-CoV-2 S2	0.58170583	0.6504653	1			
	D42 CD4+ SARS-CoV-2 S2	0.51918751	0.47326203	0.6075037	1		
	D56 CD4+ SARS-CoV-2 S2	0.05912932	0.04398827	0.34522377	0.51920341	1	
	D182 CD4+ SARS-CoV-2 S2	0.23413772	0.18083004	0.30708181	0.38522589	0.04945055	1
P value	D0 CD4+ SARS-CoV-2 S2	NA					
	D14 CD4+ SARS-CoV-2 S2	3.26E-05	NA				
	D28 CD4+ SARS-CoV-2 S2	0.00359574	1.31E-05	NA			
	D42 CD4+ SARS-CoV-2 S2	0.01898251	0.00540799	8.52E-05	NA		
	D56 CD4+ SARS-CoV-2 S2	0.79903597	0.81106632	0.03377386	0.00099194	NA	
	D182 CD4+ SARS-CoV-2 S2	0.40095888	0.40896996	0.11921927	0.04722083	0.80650014	NA

Supplementary table 7c: Spearman's correlations and P values for proliferative CD8+ T cell response to SARS-CoV-2 S1

		D0 CD8+ SARS-CoV-2 S1	D14 CD8+ SARS-CoV-2 S1	D28 CD8+ SARS-CoV-2 S1	D42 CD8+ SARS-CoV-2 S1	D56 CD8+ SARS-CoV-2 S1	D182 CD8+ SARS-CoV-2 S1
Spearman's correlation	D0 CD8+ SARS-CoV-2 S1	1					
	D14 CD8+ SARS-CoV-2 S1	0.65384615	1				
	D28 CD8+ SARS-CoV-2 S1	0.05454545	0.37979753	1			
	D42 CD8+ SARS-CoV-2 S1	0.28181818	0.61986873	0.54022397	1		
	D56 CD8+ SARS-CoV-2 S1	-0.07142857	0.36083863	0.46657929	0.3640855	1	
	D182 CD8+ SARS-CoV-2 S1	0.00606061	0.29652642	0.16695652	0.11538462	0.29774436	1
P value	D0 CD8+ SARS-CoV-2 S1	NA					
	D14 CD8+ SARS-CoV-2 S1	0.01534852	NA				
	D28 CD8+ SARS-CoV-2 S1	0.88103618	0.03843964	NA			
	D42 CD8+ SARS-CoV-2 S1	0.4011449	0.0002589	0.00067144	NA		
	D56 CD8+ SARS-CoV-2 S1	0.87904819	0.07638115	0.00814787	0.04405736	NA	
	D182 CD8+ SARS-CoV-2 S1	0.98674291	0.18023947	0.43553811	0.58284984	0.20231999	NA

Supplementary table 7d: Spearman's correlations and P values for proliferative CD8+ T cell response to SARS-CoV-2 S2

		D0 CD8+ SARS-CoV-2 S2	D14 CD8+ SARS-CoV-2 S2	D28 CD8+ SARS-CoV-2 S2	D42 CD8+ SARS-CoV-2 S2	D56 CD8+ SARS-CoV-2 S2	D182 CD8+ SARS-CoV-2 S2
Spearman's correlation	D0 CD8+ SARS-CoV-2 S2	1					
	D14 CD8+ SARS-CoV-2 S2	0.01176471	1				
	D28 CD8+ SARS-CoV-2 S2	0.03296703	0.67325617	1			
	D42 CD8+ SARS-CoV-2 S2	0.08241758	0.82495765	0.50358974	1		
	D56 CD8+ SARS-CoV-2 S2	-0.28333333	0.58668677	0.31586151	0.69379382	1	
	D182 CD8+ SARS-CoV-2 S2	0.66666667	0.02197802	-0.12745098	-0.00294118	-0.15011042	1
P value	D0 CD8+ SARS-CoV-2 S2	NA					
	D14 CD8+ SARS-CoV-2 S2	0.96550817	NA				
	D28 CD8+ SARS-CoV-2 S2	0.91485647	0.00059447	NA			
	D42 CD8+ SARS-CoV-2 S2	0.78895093	2.32E-06	0.00872244	NA		
	D56 CD8+ SARS-CoV-2 S2	0.46003033	0.00654538	0.10154498	8.47E-05	NA	
	D182 CD8+ SARS-CoV-2 S2	0.04986723	0.94318624	0.62593015	0.9913748	0.57896618	NA

Supplementary table 8: Spearman's correlations and P values for a panel of coronavirus antibody responses measured using MSD technology.

		D0 SARS-CoV-2 S	D0 SARS-CoV-1 S	D0 MERS-CoV S	D0 HKU-1 S	D0 OC43 S	D0 299E S	D0 NL63 S
Spearman's correlation	D0 SARS-CoV-2 S	1						
	D0 SARS-CoV-1 S	0.550054618	1					
	D0 MERS-CoV S	0.589027098	0.739252995	1				
	D0 HKU-1 S	0.440184644	0.461451727	0.451162791	1			
	D0 OC43 S	0.040663871	0.332346723	0.45961945	0.520366455	1		
	D0 299E S	0.159343176	0.256659619	0.146723044	0.40944327	0.469203665	1	
	D0 NL63 S	0.22854928	0.296969697	0.167159972	0.614235377	0.456095842	0.52572234	1
P value	D0 SARS-CoV-2 S	NA						
	D0 SARS-CoV-1 S	0.000109783	NA					
	D0 MERS-CoV S	2.60E-05	9.98E-09	NA				
	D0 HKU-1 S	0.002788824	0.001617085	0.002114079	NA			
	D0 OC43 S	0.79326225	0.027513953	0.00169716	0.000293719	NA		
	D0 299E S	0.301531675	0.092611767	0.341914575	0.005782097	0.001314138	NA	
	D0 NL63 S	0.135643947	0.050279606	0.278122749	9.21E-06	0.001861083	0.000247587	NA
